# Enhancing Multistep Reactions: Biomimetic Design of Substrate Channeling Using P22 Virus‐Like Particles

**DOI:** 10.1002/advs.202206906

**Published:** 2023-02-23

**Authors:** Yang Wang, Ekaterina Selivanovitch, Trevor Douglas

**Affiliations:** ^1^ Department of Chemistry Indiana University 800 E Kirkwood Ave Bloomington IN 47405 USA

**Keywords:** substrate channeling, cofactor regeneration, cascade reactions, multistep catalysis, modular nanoreactors, biomimetic materials, virus‐like particles

## Abstract

Many biocatalytic processes inside cells employ substrate channeling to control the diffusion of intermediates for improved efficiency of enzymatic cascade reactions. This inspirational mechanism offers a strategy for increasing efficiency of multistep biocatalysis, especially where the intermediates are expensive cofactors that require continuous regeneration. However, it is challenging to achieve substrate channeling artificially in vitro due to fast diffusion of small molecules. By mimicking some naturally occurring metabolons, nanoreactors are developed using P22 virus‐like particles (VLPs), which enhance the efficiency of nicotinamide adenine dinucleotide (NAD)‐dependent multistep biocatalysis by substrate channeling. In this design, NAD‐dependent enzyme partners are coencapsulated inside the VLPs, while the cofactor is covalently tethered to the capsid interior through swing arms. The crowded environment inside the VLPs induces colocalization of the enzymes and the immobilized NAD, which shuttles between the enzymes for in situ regeneration without diffusing into the bulk solution. The modularity of the nanoreactors allows to tune their composition and consequently their overall activity, and also remodel them for different reactions by altering enzyme partners. Given the plasticity and versatility, P22 VLPs possess great potential for developing functional materials capable of multistep biotransformations with advantageous properties, including enhanced efficiency and economical usage of enzyme cofactors.

## Graphical abstract

Functionally coupled enzymes and their cofactor (nicotinamide adenine dinucleotide) are colocalized inside P22 virus‐like particles. The resultant modular nanoreactors induce substrate channeling, enhancing the efficiency of cascade reactions and the utility of the cofactor.

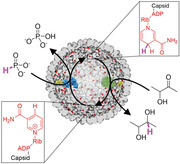

## 1 Introduction

The diversity of biocatalysis inside cells provides inspiration and a massive database for catalytic engineering, including different chemistry, ideal selectivity, and efficiency under mild conditions.^[^ ^1^, ^2^ ^]^ The cellular biocatalysts (mostly enzymes) are often functionally coupled for complex multistep cascade reactions, which usually require cofactors to assist the communication between each step to complete the overall reaction.^[^ ^3^, ^4^, ^5^ ^]^ Many cofactors are not present in the high quantities needed for each biochemical turnover in vivo, but rather a small pool of these molecules are shuttled as metabolic intermediates between enzymes and are continuously regenerated to complete mass and energy transfer.^[^ ^4^, ^6^ ^]^ To economically utilize cofactor‐dependent multistep biotransformations in vitro, biocatalytic materials are required with properties that include enhanced efficiency as well as regeneration and recycling of costly cofactors.^[^ ^2^, ^5^, ^7^ ^]^

Some naturally occurring enzyme complexes, recognized as metabolons, can achieve direct passing of intermediates from one enzymatic active site to the subsequent one.^[^ ^8^, ^9^, ^10^ ^]^ This process is defined as substrate channeling, which prevents intermediates from diffusing into bulk environment and thus increases their local concentration around the active sites.^[^ ^9^, ^10^ ^]^ As a result, multistep reactions can proceed at high overall rates even with only trace quantities of intermediates.^[^ ^9^ ^]^ With substrate channeling, metabolic flux inside cells is elevated toward desired pathways (even when enzyme concentration is very low) while toxic or labile metabolic intermediates are diminished and/or segregated from bulk environment, which assists overall cell function.^[^ ^9^, ^10^ ^]^ It was believed that colocalization of sequential enzymes would automatically lead to kinetic advantages. However, theoretical analyses demonstrate that proximity between enzymes alone does not necessarily induce substrate channeling, since most enzymes operate at rates much slower than the diffusion rate of small molecules even in a crowded space such as intracellular environment.^[^ ^8^, ^9^ ^]^ This perspective has been verified experimentally by us^[^ ^11^, ^12^ ^]^ and others^[^ ^13^, ^14^ ^]^ by packing enzymes of metabolic pathways inside compartments, with the degree of crowding close to levels found in intracellular environments and crystal packing.^[^ ^12^ ^]^ Computational modeling also suggests a mechanism whereby orientation rather than just proximity between enzymes is required to induce channeling and there is minimal effect of channeling when the active sites are over 1 nm apart,^[^ ^15^ ^]^ which is hard to achieve even in the solid state.^[^ ^12^ ^]^ This issue presents a technical challenge in designing artificial metabolons with enhanced activities.

**Figure 1:**
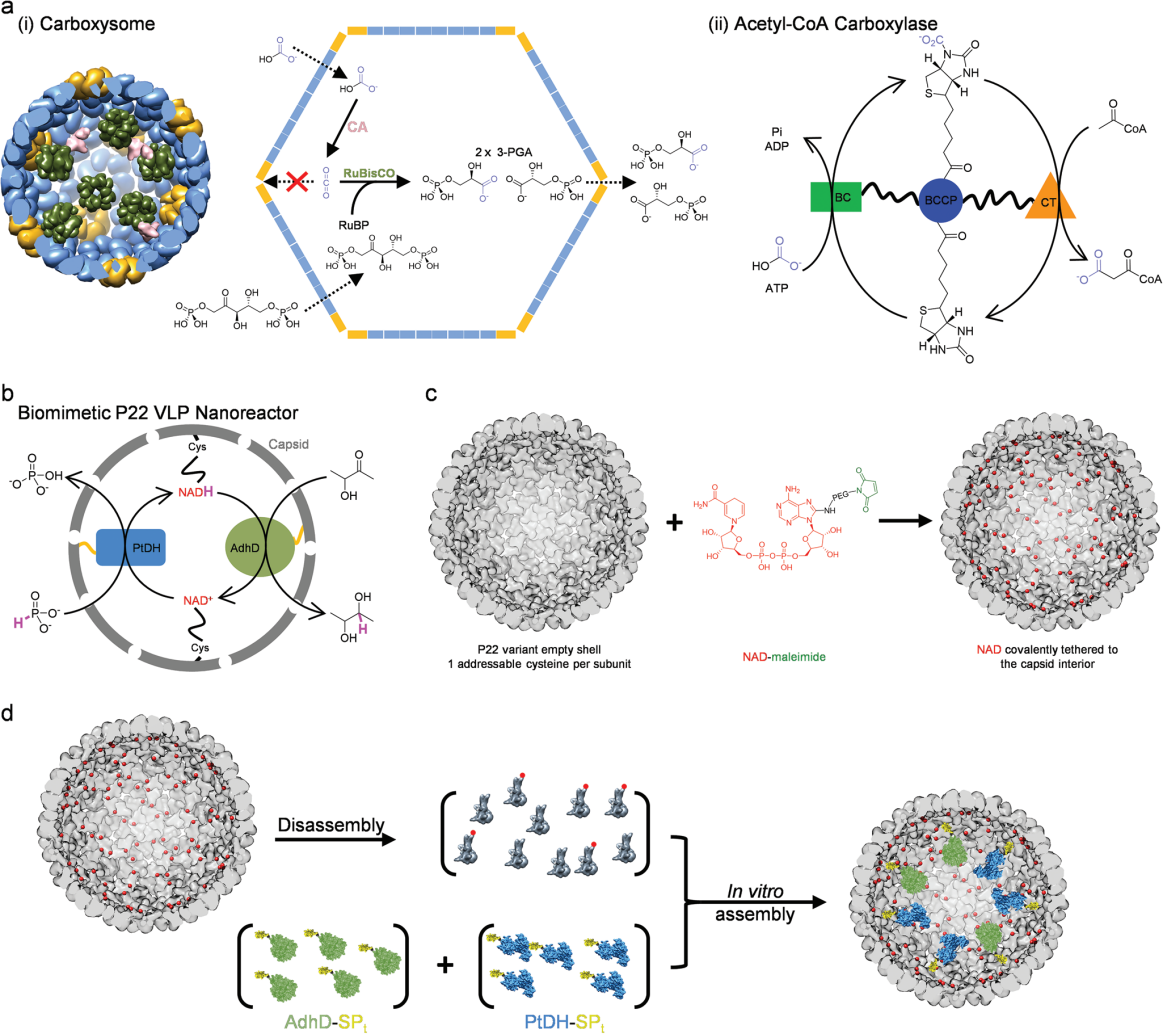
Bioinspiration for and biomimetic design of P22 VLP nanoreactors capable of an enhanced multistep reaction. a) i) Carboxysome catalyzes the two‐step carbon fixation. Multiple copies of carbonic anhydrase (CA) and ribulose‐1,5‐bisphosphate carboxylase/oxygenase (RuBisCO) are colocalized inside the protein compartment (left; this is only a model representation and does not reflect the actual scenario in carboxysome; PDB: 7CKC, 7ZBT, 2FGY). Due to selective permeability of the capsid, CO_2_ produced by CA is prevented from diffusing out of the capsid, which increases the local concentration of the intermediate close to RuBisCO and induces channeling (right). ii) Acetyl‐CoA carboxylase catalyzes a two‐step carboxyl transfer reaction. The cofactor, biotin, is covalently attached to biotin carboxyl carrier protein (BCCP) domain by a swing arm and shuttled between biotin carboxylase (BC) domain and carboxylase transferase (CT) domain, which induces channeling of the reaction intermediate. b) In this biomimetic nanoreactor, two functionally coupled enzymes, PtDH and AdhD, are encapsulated inside the P22 VLP, and their cofactor, NAD, is covalently tethered to the VLP interior by a swing arm. P22 VLP provides a colocalization environment while the swing arm limits the diffusion of the cofactor intermediate. c) Covalent immobilization of NAD is realized by bioconjugation between engineered P22 VLP and NAD‐maleimide (the reduced form is shown in the structure). d) The biomimetic P22 nanoreactor can be realized by in vitro assembly.

Investigations into metabolons show that nature has evolved strategies to realize substrate channeling by controlling diffusion of small molecule intermediates, either by guiding the direction of diffusion (such as molecular tunnels and electrostatic guidance) or by limiting the space for diffusion (such as covalent tethering and spatial confinement).^[^ ^9^ ^]^ For example, some metabolons comprise clearly defined noncatalytic structures that are solely for controlling the spatial range of intermediate diffusion. In carboxysomes, the noncatalytic capsid proteins form a compartment with a cage‐like architecture and selective permeability, which not only defines a space for colocalizing an enzyme couple noncovalently but also limits the diffusion of the intermediate (CO_2_) inside the compartment to maintain a high local concentration (**Figure** **1**ai).^[^ ^16^ ^]^ This mechanism facilitates substrate channeling and the overall anabolic pathway of carbon fixation.^[^ ^16^ ^]^ In a contrasting example of acetyl‐CoA carboxylase, the noncatalytic biotin carboxyl carrier protein domain possesses a flexible swing arm that covalently tethers the enzyme cofactor.^[^ ^17^ ^]^ With this structure, the cofactor intermediate is constrained from diffusing into the bulk solution and is instead channeled between the catalytic domains for regeneration, enhancing the efficiency of the two‐step carboxyl transfer catalysis (Figure 1aii).^[^ ^18^ ^]^

Using virus‐like particles (VLPs) derived from *Salmonella typhimurium* bacteriophage P22, we have designed biomimetic, modular metabolons capable of enzymatic cascade reactions with enhanced efficiency. This is achieved by substrate channeling between functionally coupled enzyme partners and concomitant in situ regeneration of their shared cofactor, nicotinamide adenine dinucleotide (NAD). P22 VLP is a 56 nm, *T* = 7, icosahedral capsid, which results from self‐assembly of 420 copies of coat proteins (CPs) directed by ≈100–300 copies of scaffold proteins (SPs).^[^ ^19^, ^20^, ^21^ ^]^ Programmed encapsulation of cargo proteins has been demonstrated by genetic fusion of cargo proteins to truncated SP (SP_t_),^[^ ^22^ ^]^ with no changes in the behavior of VLP assembly both in vivo (recombinant coexpression)^[^ ^23^ ^]^ and in vitro (with purified proteins).^[^ ^24^ ^]^ By encapsulating enzymes, we have successfully constructed P22 VLP nanoreactors capable of a variety of biochemical reactions.^[^ ^25^, ^26^ ^]^ Previous work shows simple enzyme encapsulation inside P22 VLPs fails to induce significant kinetic advantage in multistep cascade reactions.^[^ ^11^, ^12^ ^]^ In this work, we studied multistep NAD‐dependent hydride transfer reactions catalyzed by functionally coupled enzymes (Figure 1b). An engineered phosphite dehydrogenase (PtDH)^[^ ^27^ ^]^ was chosen for regeneration of NADH given the highly favorable thermodynamics of the reaction,^[^ ^28^ ^]^ while a ketone reduction catalyzed by alcohol dehydrogenase D (AdhD), from *Pyrococcus furiosus*, was selected as a model reaction of hydride acceptance due to the potential industrial utility of alcohol dehydrogenases and their alcohol products.^[^ ^29^ ^]^ The dense packing of proteins inside P22 VLPs resulting from enzyme encapsulation (biomacromolecular concentration ≈300 mg mL^−1^) resembles the architecture of carboxysomes, inducing colocalization of the enzymes and CP.^[^ ^19^, ^30^ ^]^ Highly porous P22 capsids show no barrier to small molecule diffusion, different from the molecular discrimination exhibited by carboxysomes.^[^ ^19^, ^31^ ^]^ By mimicking the swing arm mechanism in acetyl‐CoA carboxylase, an NAD cofactor is covalently tethered to the interior side of the noncatalytic CP subunits to control its diffusion, inducing channeling of the cofactor between the two enzymes. Since the high proximity between the enzyme couple and their cofactors is maintained by noncovalent SP–CP interactions,^[^ ^19^, ^32^ ^]^ this artificial metabolon can be disassembled and tailored for other redox reactions by recycling NAD‐labeled CP subunits. This plasticity of the system was additionally demonstrated by reduction of carbon–carbon double bonds catalyzed by an ene reductase enzyme.

## 2 Results

### 2.1 Immobilization of Nicotinamide Adenine Dinucleotide on Coat Protein and Assembly of Nanoreactors

Genetic incorporation of addressable cysteines at suitable sites on the CP allows site‐specific labeling of the P22 capsid interior^[^ ^33^ ^]^ with an NAD‐maleimide derivative, given that the only endogenous cysteine (C405) of wild‐type CP is not addressable.^[^ ^20^, ^34^ ^]^ NAD has been reported to exhibit plasticity for modification on its adenine moiety with some decrease in its activity as an enzyme cofactor.^[^ ^31^, ^35^ ^]^ Based on an established method, the adenine moiety of NAD was functionalized to incorporate a maleimide group to allow conjugation with thiols (Figure S1, Supporting Information). UV–vis data indicates that about 20% of the synthesized NAD‐maleimide molecule is in reduced form (Figure S2, Supporting Information). The activity of NAD, with PtDH, decreased by about 60% after modification (Figure S3, Supporting Information), consistent with literature reports.^[^ ^36^ ^]^ The CP‐NAD bioconjugation was realized by incubation of genetically modified P22 VLP (empty shell) with NAD‐maleimide (Figure 1c). After NAD labeling, an increase in molecular weight of CP was observed by sodium dodecyl sulfate–polyacrylamide gel electrophoresis (SDS‐PAGE; Figure S4, Supporting Information). Liquid chromatography–mass spectrometry (LC‐MS) also confirmed the successful NAD‐CP conjugation (Figure S5, Supporting Information).

To obtain the designed nanoreactors conceptualized in Figure 1b, previously reported in vitro assembly methods^[^ ^26^, ^37^ ^]^ were applied to allow colocalization of PtDH and AdhD inside NAD‐modified P22 VLPs (Figure 1d). NAD‐labeled VLPs were disassembled into NAD‐CP subunits. The His_6_‐tagged enzyme‐SP_t_ fusion proteins were recombinantly expressed and purified using immobilized metal affinity chromatography (Figures S6 and S28, Supporting Information). The enzyme‐SP_t_ fusions were then added to NAD‐CP subunits, which initiated the assembly process. Assembled VLP nanoreactors were isolated and characterized by SDS‐PAGE, size exclusion chromatography coupled with multi‐angle and quasi‐elastic light scattering (SEC‐MALS‐QELS) and transmission electron microscopy (TEM). (The data are presented later for individual assemblies.) The results show that AdhD and PtDH coassembled with NAD‐CP to form VLP nanoreactors with high enzyme packing density, as seen in reported P22 VLP systems.^[^ ^12^, ^19^, ^26^, ^37^ ^]^ The particles were intact and homogenous with a size and morphology similar to wild‐type VLP in the procapsid (PC) morphology.

### 2.2 Enhancement of Multistep Efficiency by Substrate Channeling

**Figure 2:**
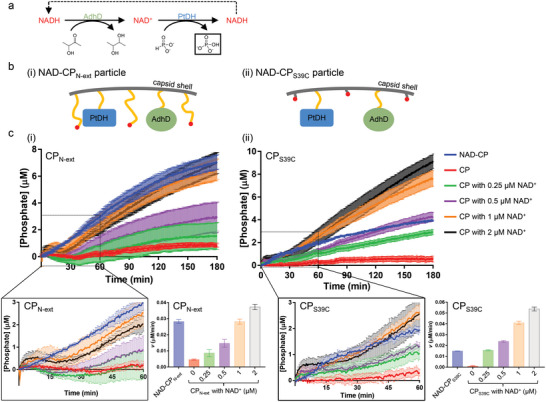
Activity of the biomimetic P22 VLP nanoreactors. a) A linear scheme of the two‐step hydride transfer reaction. Phosphate is depicted as the final product. b) The length of the swing arm is longer in i) NAD‐CP_N‐ext_ than ii) NAD‐CP_S39C_, which likely influences the accessibility of the immobilized NAD to the encapsulated enzymes. c) Kinetic progression of phosphate production for i) CP_N‐ext_ particles and ii) CP_S39C_ particles (mean ± s.e.m., *n* = 3). The early stage (0–60 min) of the reaction is zoomed in. The rate of the steady state phase is estimated by the average velocity between 90 and 180 min, displayed in the histograms.

The two domains of the NAD‐maleimide molecule are connected by a linker consisting of 10 polyethylene glycol (PEG) segments, which can serve as a flexible “swing arm” after NAD immobilization; the net length of this PEG linker is estimated to be about 4 nm when it is fully extended.^[^ ^38^ ^]^ This implies that the NAD immobilized on the VLP inner surface is likely to only sample the volume within about 4 nm from the capsid surface (probably even less when considering the linker conformation). Previous cryo‐electron microscopy (cryo‐EM) data show that the encapsulated cargo proteins are anchored to the PC shell via CP‐SP interactions and form a more than 10 nm thick shell,^[^ ^39^ ^]^ which is probably beyond the region where the immobilized NAD can access. This limited access of the NAD might reduce the availability of the cofactor to the active sites of the encapsulated enzymes, resulting in lower utility level of the immobilized NAD and consequently reaction efficiency. Therefore, we employed two different CP variants to investigate the effects of the swing arm length on nanoreactor activity (**Figure** **2**b). A previously reported single S39C substitution (CP_S39C_; Figure S7, Supporting Information) allows molecular conjugation to the inner surface.^[^ ^33^ ^]^ Additionally, a new CP variant (CP_N‐ext_) was designed with a long extension of N‐terminus, which, from the cryo‐EM structure,^[^ ^21^ ^]^ is likely located inside the capsid (Figure S7, Supporting Information), with a cysteine incorporated at the end of the terminus (see Supporting Information for sequences and design detail). The extension is about 50‐amino acid long (≈17–20 nm when fully extended^[^ ^40^ ^]^), containing Gly‐Ser repeats and a part of SP. CP_N‐ext_ has similar behavior to the wild‐type with respect to particle formation and characteristics (Figure S8, Supporting Information). This strategy provides a simple means to increase the length of the swing arm and the flexibility of immobilized NAD without changing the synthetic process of NAD‐maleimide coupling (Figure 2b). The in vitro assembled particles were characterized (Figures S9–S11, Supporting Information), and, notably, the percentage of NAD‐CP in the whole CP population is similar between CP_S39C_ (36%) and CP_N‐ext_ (38%; Figure S9, Supporting Information).

Kinetic studies on the two‐step reaction, catalyzed by the encapsulated AdhD and PtDH, were performed by monitoring phosphate production (Figure 2a). The kinetic data in Figure 2c show that NAD‐CP particles (both NAD‐CP_N‐ext_ and NAD‐CP_S39C_) catalyzed phosphate formation, suggesting immobilized NAD shuttles between the encapsulated enzymes to assist the two‐step hydride transfer, in contrast to the enzyme‐encapsulated particles assembled from unlabeled CP. To better understand the kinetic behavior of NAD‐CP particles, the unlabeled CP particles were supplemented with unmodified NAD^+^ that can freely diffuse in solution and through the capsid shell. (NAD^+^ was used rather than NADH because over 80% of NAD‐maleimide was already in the oxidized form before coupling with CP.) In the early stages (0–60 min) of the reaction, a lag phase in phosphate production was observed for NAD^+^‐supplemented unlabeled CP particles before reaching the steady‐state rate, while for NAD‐CP_N‐ext_ particles the lag phase was minimal and the phosphate production rate remained almost constant through the kinetic progression. This stark contrast is indicative of substrate channeling in the multistep cascade reaction catalyzed by the NAD‐CP particles.^[^ ^9^ ^]^

We also observed a burst phase (about 0–5 min in the kinetic plots) before the lag phase for free NAD^+^‐supplemented unlabeled CP particles, which became more prominent with increasing NAD^+^ concentration (Figure 2c). The burst phase diminished when NAD^+^ was replaced by NADH (Figure S29, Supporting Information). Therefore, this phenomenon may be partially attributed to the initial rapid reduction of NAD^+^ by PtDH. When only the oxidized form of NAD was added, the reduction of NAD^+^ by PtDH achieved its highest rate initially and then started to slow down as NAD^+^ was converted to NADH. In contrast the oxidation rate of NADH by AdhD started from zero (no NADH) and increased with the increasing NADH:NAD^+^ ratio until reaching steady state. At the end of the burst phase, it is likely that, due to decreased NAD^+^ concentration, the initial fast reduction of supplemented NAD^+^ slowed down, and the phosphate production gradually started to reflect the overall cascade reaction. Simultaneously, the NADH:NAD^+^ ratio was still lower than the value required in the steady state (Table S1, Supporting Information), suggesting that steady state had not been reached at the end of the burst phase and a lag phase should be expected (which was observed).

To evaluate if the channeling event offered a higher overall multistep rate, the velocity of the steady state (90–180 min) was calculated (Figure 2c). In the NAD‐CP nanoreactors, the total concentration of NAD in the entire solution (contributed only by the immobilized NAD) was about 0.3 × 10^−6^ m (Figure S9, Supporting Information), and the apparent activity of immobilized NAD on NAD‐CP_N‐ext_ particles was equivalent to that of approximately 1 × 10^−6^ m unmodified NAD^+^ (Figure 2ci), i.e., the local NAD concentration that is effective to the enzymes was about 3–4‐fold higher than the total NAD concentration in solution. Given that the endogenous activity of NAD decreased by about 60% after modification (Figure S3, Supporting Information), colocalization with the enzymes inside NAD‐CP_N‐ext_ particles raised the effectiveness of the modified NAD by more than fivefold. This shows that, at the same small quantity of NAD, covalent tethering of NAD on NAD‐CP_N‐ext_ VLP nanoreactors made the two‐step reaction more efficient than the reaction with free NAD.

The kinetic data also show the influence of the swing arm on the effectiveness of the immobilized NAD to the encapsulated enzyme couple. In contrast to the increase observed in NAD‐CP_N‐ext_ particles (Figure 2ci), the apparent activity of the immobilized NAD on NAD‐CP_S39C_ particles was only equivalent to about 0.25 × 10^−6^ m of free unmodified NAD^+^ (Figure 2cii), which is similar to the total NAD concentration in solution (about 0.3 × 10^−6^ m; Figure S9, Supporting Information). These results suggest that the length of swing arm can affect the multistep efficiency, likely due to different accessibility of the immobilized cofactor to the proximate enzymes.

### 2.3 Tuning Multistep Efficiency by Controlling Assembly Composition

One of the advantages of the P22 in vitro assembly system is that the ratio of input components can be easily adjusted to alter the composition of the assembled particle as a means to assess the effects of stoichiometry on the behavior of the particles.^[^ ^26^, ^37^ ^]^ This flexibility was employed to study how the quantity of immobilized NAD and the ratio between the two functionally coupled enzymes influence the activity of the P22 VLP nanoreactors.

**Figure 3:**
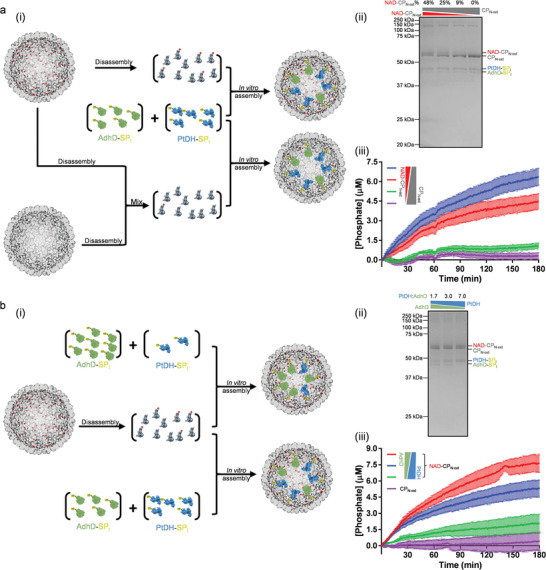
Tuning the composition of the VLP nanoreactors. a) i) The quantity of immobilized NAD can be tuned by mixing NAD‐CP_N‐ext_ and unlabeled CP_N‐ext_ during in vitro assembly. ii) SDS‐PAGE shows the percentage of NAD‐CP_N‐ext_ is successfully altered, where the percentage is estimated by densitometry analysis (Figure S14, Supporting Information). iii) VLP nanoreactors with more NAD loading exhibits higher kinetic efficiency of the catalysis of the two‐step reaction, determined by phosphate production (mean ± s.e.m., *n* = 3). b) i) The enzyme ratio in the nanoreactors can be tuned by altering enzyme stoichiometry during in vitro assembly. ii) SDS‐PAGE shows enzyme ratio is successfully altered among different assemblies, where the AdhD:PtDH ratio is estimated by densitometry analysis (Figure S17, Supporting Information). iii) The enzyme ratio affects the multistep activity of the nanoreactors, determined by phosphate production (mean ± s.e.m., *n* = 3).

We hypothesized that more NAD immobilized on the VLP would result in access of more NAD to the encapsulated enzymes and consequently a higher overall activity of the nanoreactors. Addition of unlabeled CP together with NAD‐CP during the in vitro assembly allows us to control the number of NAD‐CP in the assembled VLP and thus the amount of NAD immobilized on the particle (**Figure** **3**ai). The morphology and homogeneity of the particles were similar (Figures S12 and S13, Supporting Information), for particles where NAD‐CP_N‐ext_:unlabeled CP_N‐ext_ ratio was tuned in a controlled manner (Figure 3aii; Figure S14, Supporting Information). Kinetic analysis shows that the activity of VLP nanoreactors was the highest for VLPs with the highest NAD immobilization (Figure 3aiii). The activity of the immobilized NAD inside the particles was also compared to unmodified NAD^+^ (Figure S15, Supporting Information), which demonstrates a positive correlation between the activity of nanoreactors and amount of immobilized NAD.

The overall efficiency of a multistep reaction is usually determined by the rate of the slow step. For enzymatic multistep reactions, the rate of each step is determined not only by the catalytic properties of enzymes but also their concentrations. Some naturally occurring systems, such as *Bacillus subtilis* lumazine synthase‐riboflavin synthase complex,^[^ ^41^ ^]^ appear to maximize overall efficiency of pathways by balancing the rates of individual steps, which is realized by optimization of the relative stoichiometry of the enzymes. Since the catalytic rate of PtDH is much higher than AdhD (about 40‐fold; Figure S16, Supporting Information), changing enzyme stoichiometry may enhance the overall multistep efficiency of the NAD‐CP_N‐ext_ VLP nanoreactor by balancing the rates of the two coupled reactions. By altering the input enzyme ratio in the in vitro assembly (Figure 3bi), the enzyme composition of the particles was tuned (Figure 3bii; Figure S17, Supporting Information) without change in particle formation (Figures S18 and S19, Supporting Information). The kinetic data shows that the activity of the nanoreactors changed with different enzyme stoichiometry (Figure 3biii). Within the range of the tested enzyme stoichiometry, the overall efficiency was the highest when the slower enzyme (AdhD) was present at the highest amount, i.e., when the two steps of the cascade reaction exhibited the closest rates. This suggests the catalytic rates of the two enzymes need to be balanced for the optimized continuous regeneration of NADH and the maximized activity of the nanoreactor.

### 2.4 Functional and Modular Nanomaterials for Enzymatic Multistep Reactions

**Figure 4:**
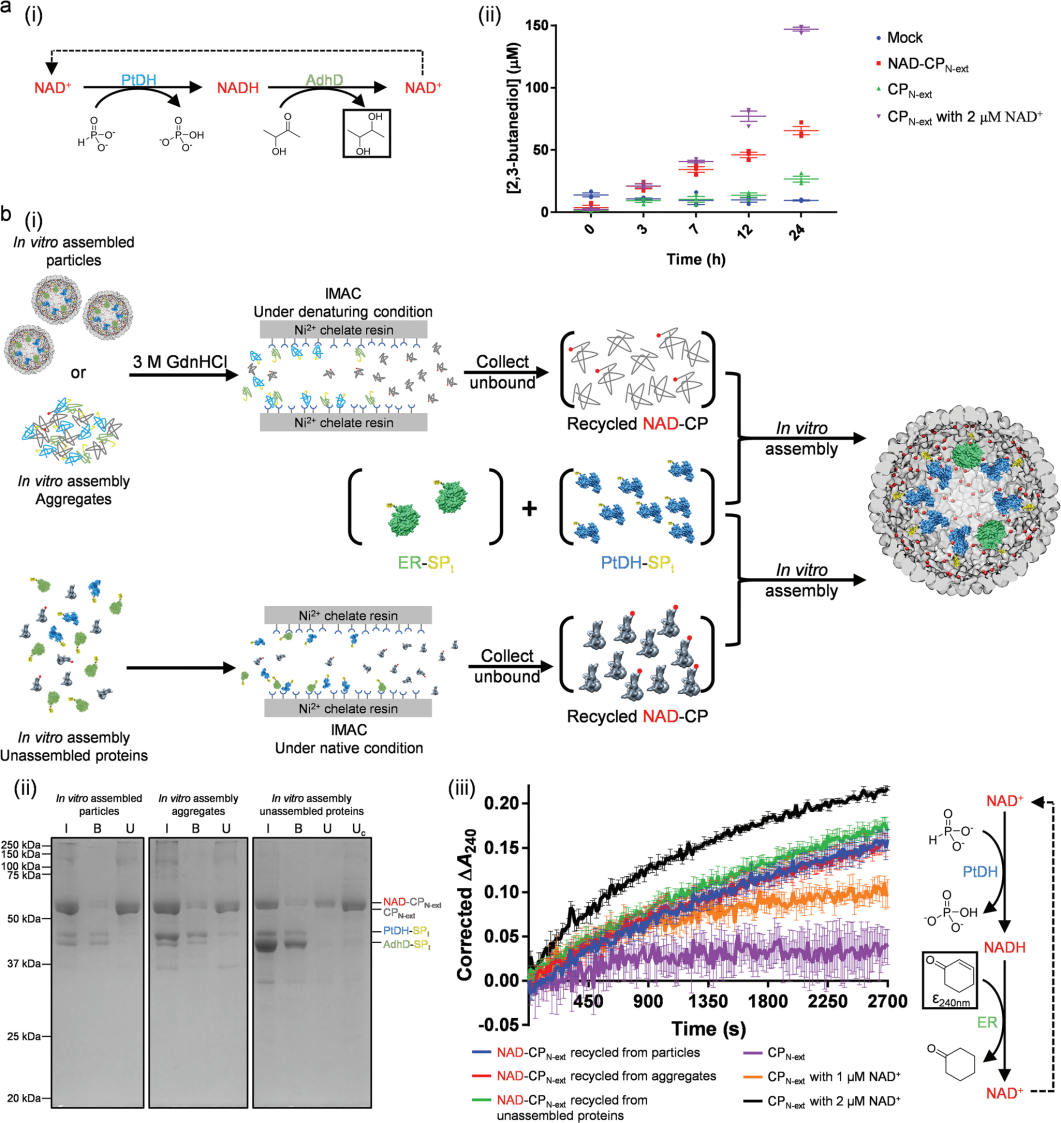
The biomimetic P22 VLP nanoreactors as functional and modular biocatalytic materials. a) i) The production of 2,3‐butanediol is investigated as the final product of the two‐step reaction. ii) The NAD‐CP_N‐ext_ particles showed continuous biocatalytic activity for 24 h (mean ± s.e.m., *n* = 3). b) i) NAD‐CP_N‐ext_ can be recycled from in vitro assembled particles and the unassembled proteins during in vitro assembly, for remodeling the nanoreactors for another two‐step hydride transfer reaction catalyzed by PtDH and an ene reductase (ER). ii) SDS‐PAGE shows that incubation with Ni^2+^ chelate resin removed most of the His‐tagged enzymes from NAD‐CP_N‐ext_ (I = input, i.e., proteins before incubation with His‐tag affinity resin; B = resin‐bound fraction; U = unbound fraction; U_c_ = concentrated unbound fraction; U and U_c_ were used for the new round of in vitro assembly). iii) The new nanoreactors are active in catalysis of the reduction of carbon–carbon double bond in cyclohexenone (mean ± s.e.m., *n* = 3).

To investigate whether the biomimetic VLP nanoreactors can be used as functional biocatalytic materials, we also monitored 2,3‐butanediol, the product of the AdhD reaction (**Figure** **4**ai), which is an important industrial precursor and proposed source of biofuel.^[^ ^42^ ^]^ Using gas chromatography–mass spectrometry (GC‐MS), we were able to confirm the synthesis of 2,3‐butanediol (Figure S20, Supporting Information). Quantitative analysis suggests the immobilized NAD clearly exhibited activity continuously over a 24 h period (Figure 4aii; Figures S21 and S22, Supporting Information; consistent with the results in Figure 2ci which shows the effective concentration of immobilized NAD is about 1 × 10^−6^ m), and was recycled over 200 times, demonstrating the robustness of the system.

In our design, proximity is introduced between functionally coupled enzymes and their cofactor using the noncovalent interactions between CP and SP. The modular feature of P22 allows us to easily disassemble the particles and recycle NAD‐CP for remaking active nanoreactors after the encapsulated enzymes are beyond their lifetime (especially when they are prone to inactivation), or for remodeling the nanoreactors for other enzymatic chemistry. The initial particle assembly yield is only 10%–20% using the current (unoptimized) in vitro assembly protocol,^[^ ^24^, ^26^, ^37^ ^]^ and a lot of input proteins remain unassembled or form aggregates. Recycling NAD‐CP from the unassembled components allows for a new round of in vitro assembly, circumventing the drawback of low assembly yield.

We show here that new nanoreactors can be easily made using NAD‐CP_N‐ext_ recovered and recycled from the nanoreactors for ketone reduction described above, as well as the unassembled components after the initial in vitro assembly (Figure 4bi). We formed new VLPs by coassembly of PtDH, an ene reductase (ER from *Lactobacillus casei*;^[^ ^43^ ^]^ Figure S30, Supporting Information), and the recycled NAD‐CP_N‐ext_ (Figure 4bii). The resultant VLP nanoreactors (Figure S23–S25, Supporting Information) incorporated active PtDH and ER enzymes and were capable of carbon–carbon double bond reduction (Figure 4biii; Figure S26, Supporting Information). The activity of immobilized NAD was not compromised by the recycling process and was determined to still be equivalent to (1–2) × 10^−6^ m unmodified (free) NAD^+^ (Figure 4biii), which is similar to what we observed in the ketone reduction (Figure 2ci). These results demonstrate that NAD‐CP VLP nanoreactors are modular assemblies, and the NAD‐CP module can be detached and reused so that the immobilized cofactor can be taken full advantage of within its functional lifetime.

## 3 Discussion

Here we demonstrate biomimetic nanoreactors capable of multistep enzymatic reactions with enhanced efficiency, which is induced by substrate channeling realized by colocalization of functionally coupled enzymes with their shared cofactor inside P22 VLPs. As protein‐based compartments, P22 VLP nanoreactors possess advantageous properties,^[^ ^19^ ^]^ including plasticity in genetic and chemical modifications (for cofactor immobilization),^[^ ^33^ ^]^ the ability to induce biomacromolecular proximity (for the colocalization by VLP self‐assembly and concomitant enzyme encapsulation),^[^ ^30^ ^]^ high porosity (allowing small molecule substrates and products to freely diffuse across the capsid),^[^ ^31^ ^]^ and morphological homogeneity (as nanomaterials).^[^ ^44^ ^]^ The colocalization of enzymes and cofactor in the VLP by noncovalent interactions between the cofactor‐CP module and the enzyme‐SP modules^[^ ^32^ ^]^ (rather than irreversibly immobilization of cofactors to enzymes or enzyme complexes) lends itself to modular recycling of the components. P22 nanoreactors capable of various biochemical reactions can be readily designed and made by genetic fusion of SP to different enzymes. These features make the system highly versatile for tailoring to the needs of tunable composition as well as a range of chemistries.

Substrate channeling is the mechanism behind the increased multistep efficiency in our designed nanoreactors. The diffusion of the reaction intermediates (NAD cofactor, either the reduced or the oxidized form; acting as the intermediate in both steps of the parallel cascade reaction^[^ ^3^ ^]^ shown in Figure 1b) is controlled by covalent immobilization via a swing arm. This mechanism elevates the effective local NAD concentration available to the enzymes compared to the actual total NAD concentration in the solution, and consequently the NAD‐dependent cascade reactions can operate in a higher overall rate with the same amount of the cofactor. However, the immobilized NAD cofactor does not display as a high equivalent concentration to unmodified NAD as expected, based on theoretical calculation (Table 2, Supporting Information). This may indicate that the immobilized NAD was partially inactivated and/or not fully available for catalysis (discussed in the next two paragraphs). This could result in the concentration of functional NAD being much lower than the total amount of NAD immobilized on CP, which was the actual number used in equivalency estimation.

The activity of NAD, after functionalization with maleimide, was found to be reduced by about 60% compared to free unmodified NAD (Figure S3, Supporting Information). In our experiments, all the activity measurements of the immobilized NAD (modified) are compared to those of free NAD^+^ (unmodified). This biased comparison therefore underestimated the effect of NAD immobilization on the efficiency of the multistep reaction, and thus immobilization itself leads to an even more significant enhancement of the multistep efficiency than that observed in the experiments. The compromised activity of the immobilized NAD due to the structural modification can potentially be compensated for by raising the amount immobilized on the VLP (based on what was observed in Figure 3a). Additionally, because of a potentially high on‐rate resulting from the high local concentration of both enzymes and NAD inside the particles (in the millimolar range), there is a possibility to form tightly bound NAD–enzyme complexes which would render both the immobilized NAD and the enzymes less active for catalysis. In future optimization of this system, this issue could be resolved by decreasing the number of enzymes packed inside the capsids to reduce the local concentration of the enzymes.^[^ ^26^ ^]^

The availability of the immobilized NAD to the encapsulated enzymes can be influenced by the length and the flexibility of the swing arm. Compared to the short PEG swing arm in NAD‐CP_S39C_ nanoreactors, the swing arm in the NAD‐CP_N‐ext_ nanoreactors are elongated significantly, which increased the apparent activity of the immobilized NAD. Extension of CP N‐terminus provides a straightforward and economical platform to change the properties of the swing arm by genetic engineering.

Our study on the kinetic properties of the nanoreactors reflects the average behavior of the entire population. Individual heterogeneity likely occurs at several levels during the assembly process. The loading density of the enzymes, enzyme stoichiometry, and the amount of immobilized NAD may vary between individual particles. It is likely that there is an activity distribution within the population of P22 VLP nanoreactors that cannot be resolved by bulk measurements. Furthermore, even within a single particle, the encapsulated enzymes and NAD might not be distributed evenly. If immobilized NAD is not situated in a space where both of the enzyme partners are readily accessible, the conversion of NAD between its reduced and oxidized states can be slowed or even cease, so that the immobilized NAD can only partially (or even not at all) participate in the multistep reaction.

P22 VLP shows great potential in the development of functional biomaterials, especially in the area of biocatalysis.^[^ ^19^, ^44^ ^]^ Using the P22 VLP platform, this work achieves increased efficiency of multistep enzymatic reactions by controlling the diffusion and subsequent in situ regeneration of an NAD cofactor. This system could potentially be applied to a wide variety of NAD‐dependent cascade reactions, as suggested by the successful turnover of two different types of NADH‐consuming oxidoreductases, AdhD and ER. We have also previously reported that P22 VLP nanoreactors enhance enzyme stability against proteolysis and thermal denaturation, which prolongs the lifetime of the biocatalysts.^[^ ^12^, ^45^ ^]^ Moreover, simple methods have been developed to assemble higher‐order structures of P22 VLP nanoreactors to fabricate heterogenous catalysts, with benefits of tuned efficiency and substrate selectivity, as well as storage, reusability and downstream separation in catalytic handling.^[^ ^19^, ^46^ ^]^ Given the versatile utility of P22 VLP nanoreactors and potential accommodation of the swing arm‐based strategy to other cofactors (such as adenosine triphosphate, flavin adenine dinucleotide, and coenzyme A), the prospect of more complex functional materials can be expected for completing a broad range of biochemical pathways with enhanced multistep efficiency and more economical usage of enzyme cofactors, as well as other properties of interest such as heterogenous catalysis.

## Conflict of Interest

The authors declare no conflict of interest.

## Supporting information

Supporting information

## Data Availability

The data that support the findings of this study are available from the corresponding author upon reasonable request.
