# Supplementary material for: Enhancing Multistep Reactions: Biomimetic Design of Substrate Channeling Using P22 Virus‐Like Particles
Source: Adv Sci (Weinh). 2023 Feb 23;10(13):2206906. doi: 10.1002/advs.202206906 (PMC10161098; doi:10.1002/advs.202206906)
Supplement: Supplementary file 1 — Supporting information [file ADVS-10-2206906-s001.pdf]

ADVANCED SCIENCE
Open Access

Supporting Information

for *Adv. Sci.*, DOI 10.1002/advs.202206906

Enhancing Multistep Reactions: Biomimetic Design of Substrate Channeling Using P22 Virus-Like Particles

*Yang Wang, Ekaterina Selivanovitch and Trevor Douglas**

Supporting Information

Enhancing Multistep Reactions: Biomimetic Design of Substrate Channeling Using P22 Virus-like Particles

Yang Wang, Ekaterina Selivanovitch, and Trevor Douglas*

Department of Chemistry, Indiana University, 800 E Kirkwood Ave, Bloomington, IN 47405, United States

*Corresponding author. Email: trevdoug@indiana.edu. ORCID ID: 0000-0002-7882-2704.

Table of Contents

Supplementary Figures (p. 1-18)
Supplementary Tables (p. 19-20)
Methods (p.21-26)
Protein Sequences (p.27-28)
References (p.29)

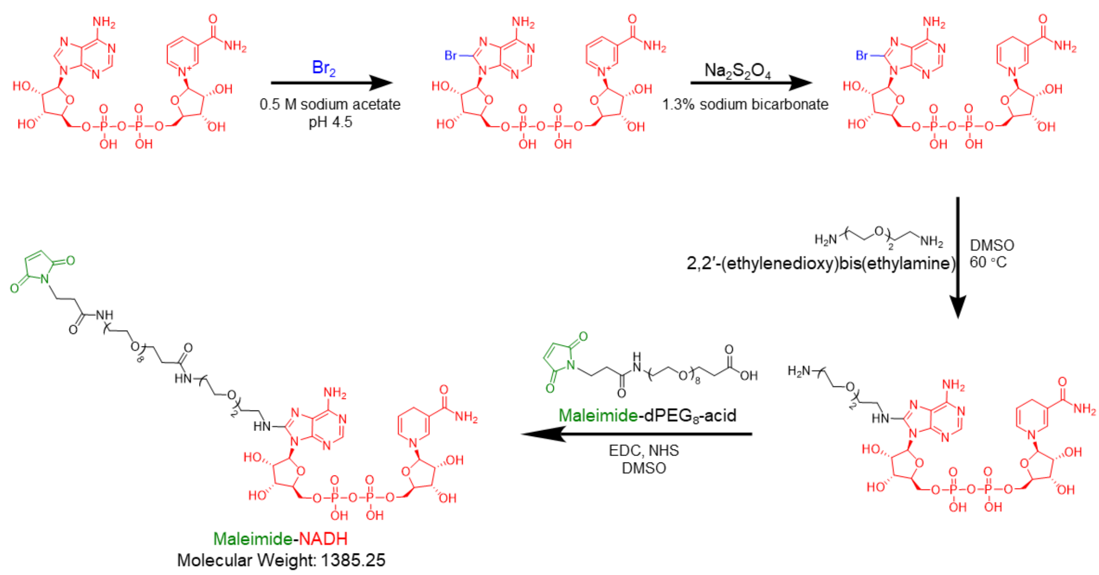

Supplementary Fig. 1. Synthetic route of NAD-maleimide.

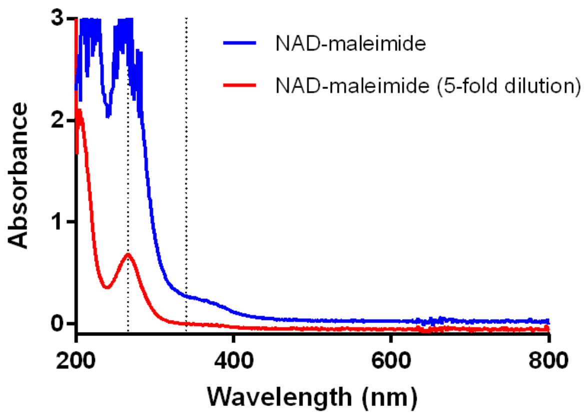

	A_{265}	A_{340}
NAD-maleimide	-	0.25095
NAD-maleimide (5-fold dilution)	0.73321	-

Supplementary Fig. 2. UV-vis spectra of NAD-maleimide (pathlength l = 1 cm).

Analysis: As the adenine moiety of NAD has been modified, its maximum absorbance shifts from 260 nm to 265 nm. From the UV-vis spectra, NAD-maleimide has absorbance A_{340} = 0.25095 and A_{265} = 0.73321 × 5 = 3.66605. The reduced form of NAD-maleimide has a concentration c_{red} = $A_{340}/(\varepsilon_{\mathrm{NADH,\ 340}}\ l)$ = 0.25095/(6.22 mM^{-1} cm^{-1} × 1 cm) = 40.346 μM, and therefore contributes to absorbance at 265 nm for $A_{265,\mathrm{red}} = \varepsilon_{\mathrm{NADH,260}}\ c_{\mathrm{red}}\ l$ = 14.4 mM^{-1} cm^{-1} × 40.346 μM × 1 cm= 0.58098. The absorbance at 265 nm contributed by the oxidized form of NAD-maleimide is $A_{265,\mathrm{ox}} = A_{265} - A_{265,\mathrm{red}}$ = 3.66605 – 0.58098 = 3.08507, and the concentration of oxidized NAD-maleimide is $c_{\mathrm{ox}} = A_{265,\mathrm{ox}}/\varepsilon_{\mathrm{NAD^+,\ 260}}$ =3.08507/(18.0 mM^{-1} cm^{-1} × 1 cm) = 171.39 μM. Therefore, reduced% = $c_{\mathrm{red}}/(c_{\mathrm{ox}} + c_{\mathrm{red}})$ = 40.346 μM/(171.39 μM + 40.346 μM) = 19%, oxidized% = 1 - reduced% = 81%. Since the NAD-CP bioconjugation and in vitro assembly processes were carried out under ambient condition, the percentage of the oxidized form is expected to be higher than 81% for the immobilized NAD in NAD-CP particles.

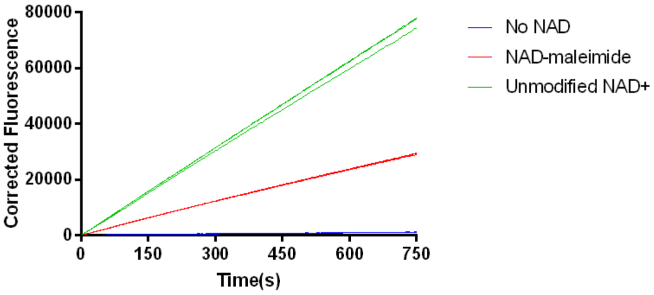

	Average Rate (ΔF/s)
No NAD	1.339 ± 0.010
NAD-maleimide	38.81 ± 0.04
Unmodified NAD$^+$	102.2 ± 0.2

Supplementary Fig. 3. Activity of NAD-maleimide molecule determined by resazurin assay (n=3, each replicate is displayed separately). Reaction condition: 10 µM NAD (when applicable), 3 mM sodium phosphite, 0.5 mM resazurin, 0.5 mM phenazine methosulfate, 2 mM 2-mercaptoethanol, 1 µM PtDH in 100 mM HEPES buffer (pH 7.2). Fluorescence was monitored using Biotek Cytation 5 plate reader (Ex: 544 nm, Em: 590 nm). NAD concentration of NAD-maleimide is determined as described in the analysis of supplementary Fig. 2.

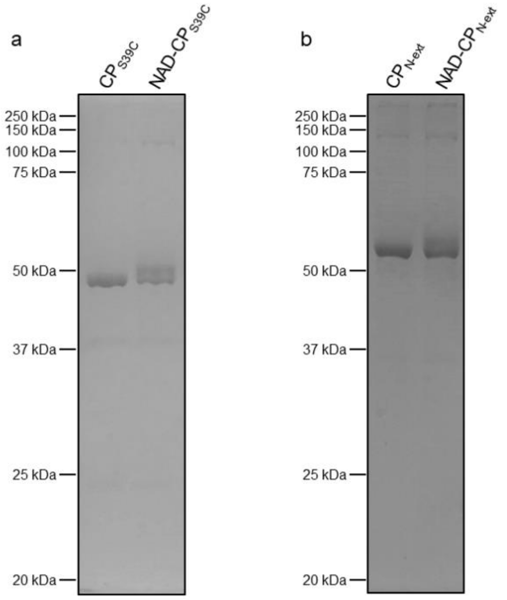

Supplementary Fig. 4. SDS-PAGE results of unlabeled CP and NAD-CP. (a) CP_{S39C}. (b) $CP_{N\text{-}ext}$. The protein bands between 100-150 kDa are likely oligomers of CP.

a

b

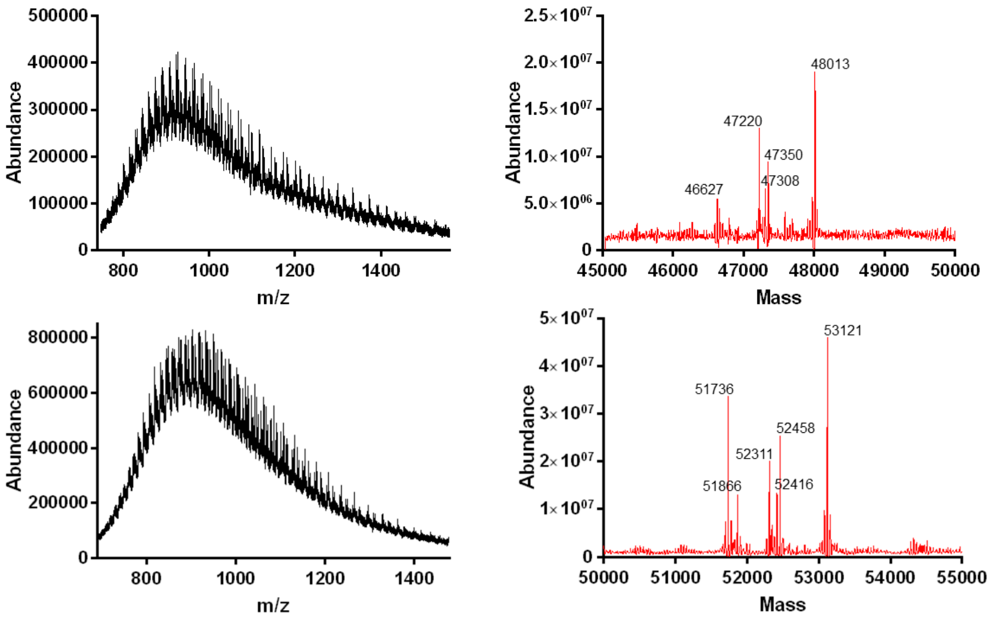

c

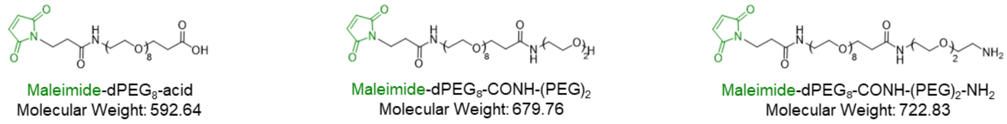

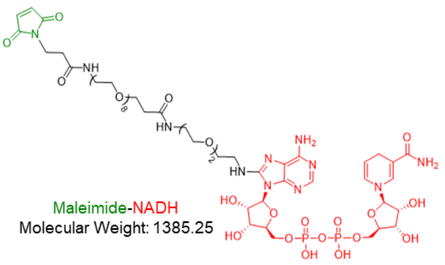

46627	Unlabeled CP_{S39C} (without methionine; expected: 46636.73)
47220 = 46627 + 593	Maleimide-dPEG$_8$-acid adduct
47308 = 46627 + 681	Maleimide-dPEG$_8$-CONH-(PEG)$_2$ adduct
47350 = 46627 + 723	Maleimide-dPEG$_8$-CONH-(PEG)$_2$-NH$_2$ adduct
48013 = 46627 + 1386	Maleimide-NADH adduct

51736	Unlabeled $CP_{N\text{-}ext}$ (without methionine; expected: 51734.96)
51866	Unlabeled $CP_{N\text{-}ext}$ (expected: 51866.15)
52311 = 51736 + 575	Maleimide-dPEG$_8$-acid adduct
52416 = 51736 + 680	Maleimide-dPEG$_8$-CONH-(PEG)$_2$ adduct
52458 = 51736 + 722 or 51866 + 592	Maleimide-dPEG$_8$-CONH-(PEG)$_2$-NH$_2$ adduct or maleimide-dPEG$_8$-acid adduct
53121 = 51736 + 1385	Maleimide-NADH adduct

Supplementary Fig. 5. LC-MS of NAD-CP. (a) NAD-CP_{S39C}. (b) NAD-$CP_{N\text{-}ext}$. (c) Analysis of the LC-MS results.

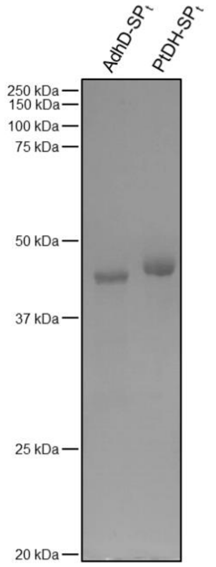

Supplementary Fig. 6. SDS-PAGE results of PtDH-SP$_t$ and AdhD-SP$_t$.

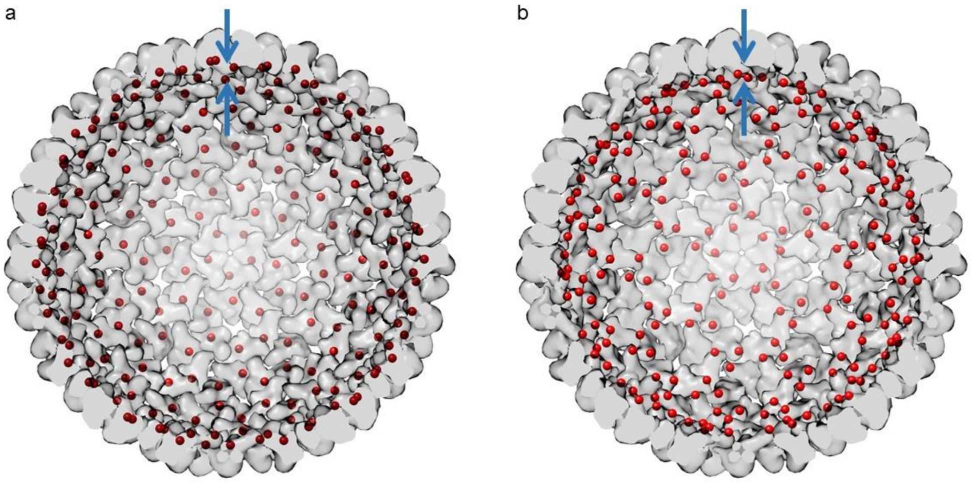

Supplementary Fig. 7. Cyro-EM structural model of P22 capsid that shows the location of the Ser39 (a) and Thr10 (b) of CP (PDB: 2XYY). Both residues are located at interior side of the P22 capsid by looking at the edge of the particle (noted in blue arrows.). The first 9 amino acid residues of CP are not resolved in the structure, likely due to the flexibility of the N-terminus.

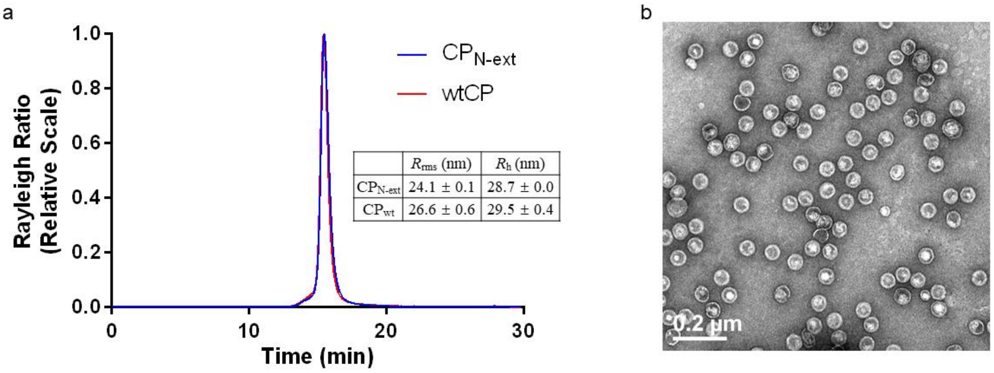

Supplementary Fig. 8. Characterization of P22 capsid assembled from $CP_{N\text{-}ext}$ by SEC-QELS (a) (mean ± s.e.m., n=3) and TEM (b).

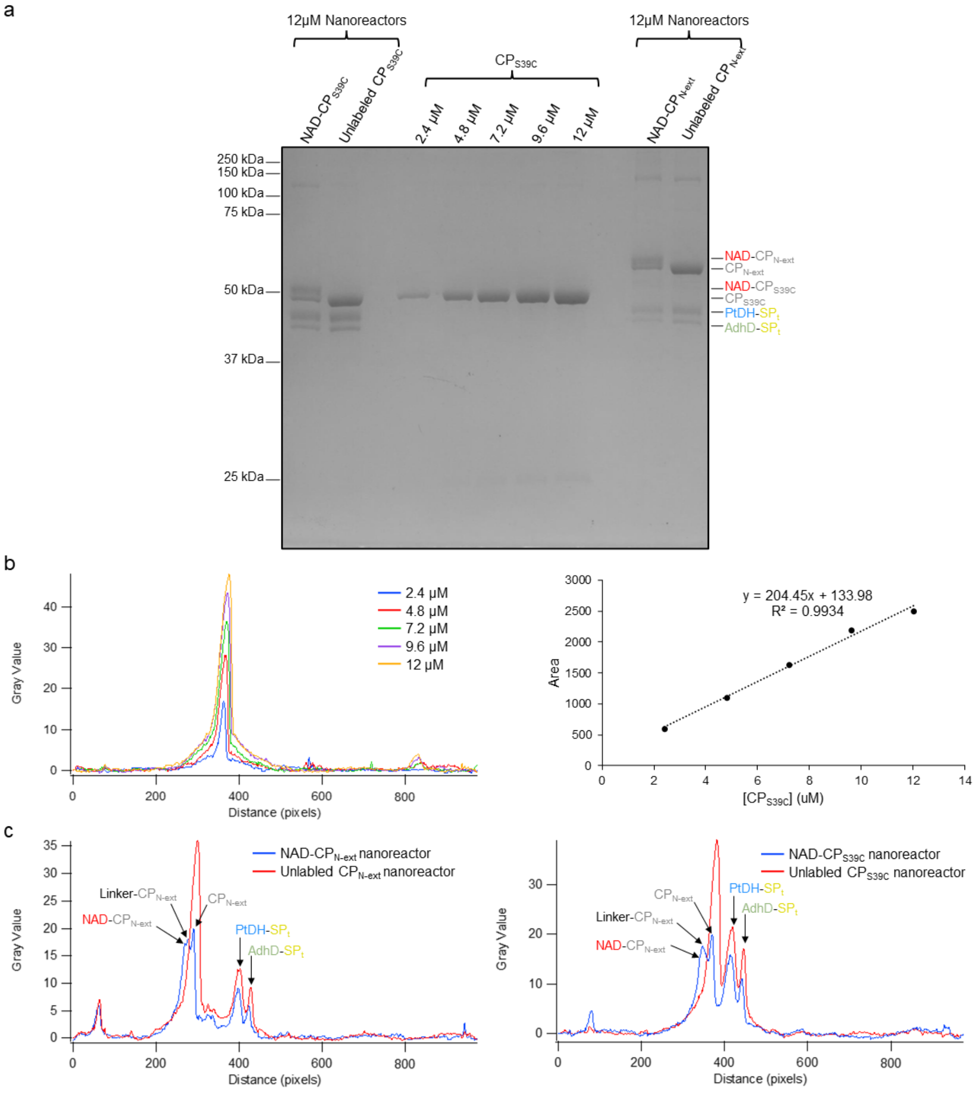

Supplementary Fig. 9. SDS-PAGE and densitometry analysis of *in vitro* assembled VLP

nanoreactors. (a) SDS-PAGE. NAD-$CP_{N\text{-}ext}$ and NAD-CP_{S39C} do not show distinct bands but smear where at least two bands can be vaguely seen, consistent with LC-MS data (supplementary Fig. 5). Concentration of CP_{S39C} standards was determined by A_{280} (ε = 44920 M^{-1} cm^{-1}). Concentration of nanoreactors was determined by Bradford Assay (CP_{S39C} as standard). (b) Densitometry plot of CP_{S39C} (left). The peak area of CP_{S39C} in the densitometry plot shows a linear response with respect to concentration (right). (c) Densitometry plot of $CP_{N\text{-}ext}$ VLP nanoreactors (left) and CP_{S39C} VLP nanoreactors (right).

Analysis: The peak areas in the densitometry plot of the NAD-$CP_{N\text{-}ext}$ band and the NAD-CP_{S39C} band were used to estimate the total concentration of NAD in the kinetic analysis of the nanoreactors. This quantification is only an estimation, since the Gaussian fitting cannot be applied due to noisy baseline and the tailing of the protein bands.

Method 1: The total CP area (NAD-CP, linker-CP, and unlabeled CP) was plugged into the equation of the calibration curve obtained in (b) to get the [CP] in the sample. Then the [NAD-CP] is calculated by multiplying [CP] by the percentage of NAD-CP area out of total CP area. This method estimates 1.70 μM NAD in 12 μM NAD-$CP_{N\text{-}ext}$ VLP nanoreactor, and 1.52 μM NAD in 12 μM NAD-$CP_{N\text{-}ext}$ VLP nanoreactor. In the kinetic study, because 1.8 μM nanoreactor is used, the total NAD concentration is estimated as 0.255 μM for NAD-$CP_{N\text{-}ext}$ VLP nanoreactor, and 0.228 μM for NAD-CP_{S39C} VLP nanoreactor.

Method 2: The [NAD-CP] in 12 μM is calculated by multiplying 12 μM by the percentage of NAD-CP area out of total protein area (including both CP and enzymes). This method estimates 3.21 μM NAD in 12 μM NAD-$CP_{N\text{-}ext}$ VLP nanoreactor and 2.40 μM NAD in 12 μM NAD-$CP_{N\text{-}ext}$ VLP nanoreactor. In the kinetic study, because 1.8 μM nanoreactor is used, the total NAD concentration is estimated as 0.481 μM for NAD-$CP_{N\text{-}ext}$ VLP nanoreactor and 0.359 μM for NAD-CP_{S39C} VLP nanoreactor. In this method, the calibration curve in (b) is not used.

From the two methods of analysis, the average of total [NAD] in the kinetic study is estimated as 0.368 μM for NAD-$CP_{N\text{-}ext}$ VLP nanoreactor and 0.294 μM for NAD-CP_{S39C} VLP nanoreactor. Note that, the total protein concentration of both CP_{S39C} and $CP_{N\text{-}ext}$ particles was determined by Bradford assay using CP_{S39C} as standard (quantified by A_{280}). (Due to nucleic acid contamination (supplementary Fig. 31), we were not able to obtain $CP_{N\text{-}ext}$ that can be quantified by A_{280}, and thus $CP_{N\text{-}ext}$ was not used as standards in Bradford assay to determine the total protein concentration of $CP_{N\text{-}ext}$ particles.) Because the N-terminal extension of $CP_{N\text{-}ext}$ increases the binding with and Coomassie stain, the concentration NAD-$CP_{N\text{-}ext}$ is overestimated. Based on this analysis, 0.3 μM is a good estimation of total [NAD] in solution in the kinetic study for both NAD-$CP_{N\text{-}ext}$ nanoreactors and NAD-CP_{S39C} particles.

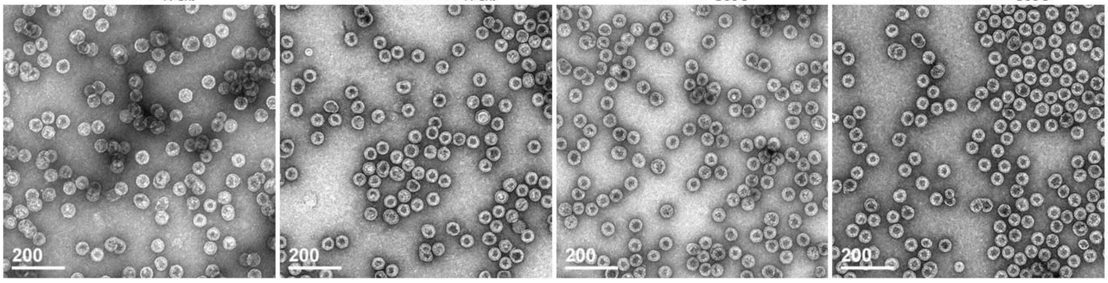

Supplementary Fig. 10. TEM images of *in vitro* assembled VLP nanoreactors. Scale bar: 200 nm.

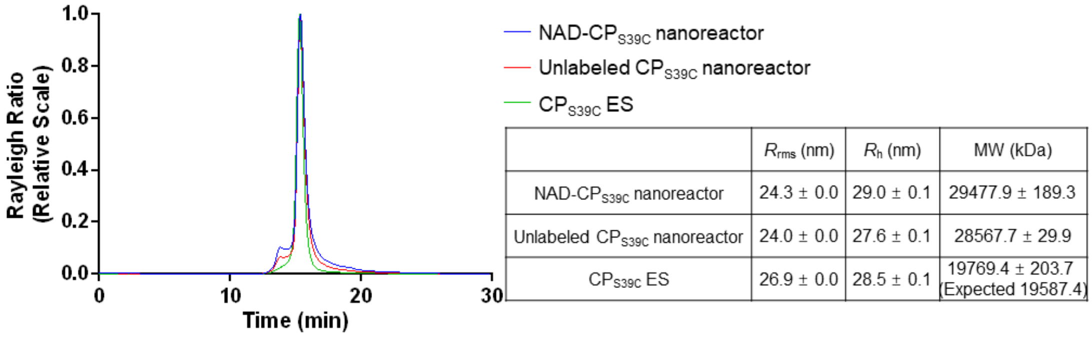

	R_{rms} (nm)	R_h (nm)	MW (kDa)
NAD-CP$_{S39C}$ nanoreactor	24.3 ± 0.0	29.0 ± 0.1	29477.9 ± 189.3
Unlabeled CP$_{S39C}$ nanoreactor	24.0 ± 0.0	27.6 ± 0.1	28567.7 ± 29.9
CP$_{S39C}$ ES	26.9 ± 0.0	28.5 ± 0.1	19769.4 ± 203.7 (Expected 19587.4)

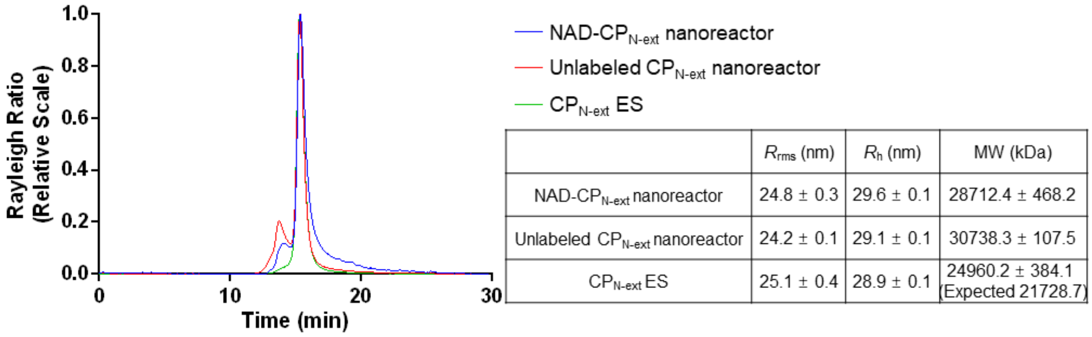

	R_{rms} (nm)	R_h (nm)	MW (kDa)
NAD-CP$_{N\text{-}ext}$ nanoreactor	24.8 ± 0.3	29.6 ± 0.1	28712.4 ± 468.2
Unlabeled CP$_{N\text{-}ext}$ nanoreactor	24.2 ± 0.1	29.1 ± 0.1	30738.3 ± 107.5
CP$_{N\text{-}ext}$ ES	25.1 ± 0.4	28.9 ± 0.1	24960.2 ± 384.1 (Expected 21728.7)

Supplementary Fig. 11. Characterization of *in vitro* assembled VLP nanoreactors by SEC-MALS-QELS (mean ± s.e.m., n=3). The molecular weights of nanoreactors increased by ~7-10 MDa compared to the expected molecular weight of ES. CP$_{N\text{-}ext}$ ES showed an molecular weight higher than expected due to nucleic acid contamination, while nucleic acid contamination is much less for CP$_{N\text{-}ext}$ nanoreactors (supplementary Fig. 31).

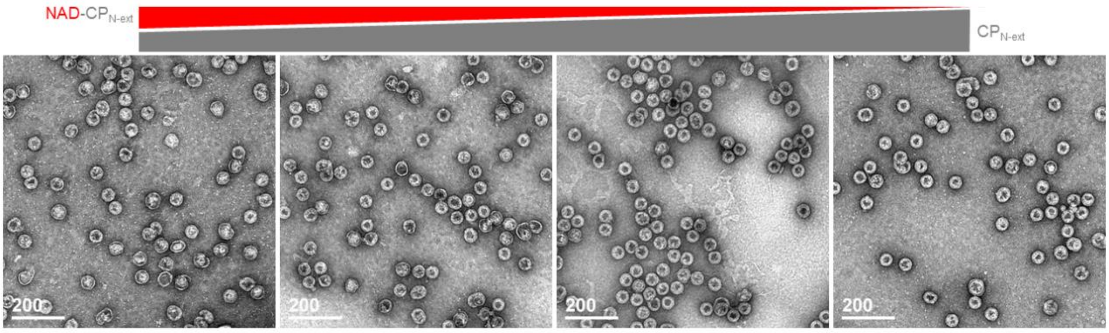

Supplementary Fig. 12. TEM images of *in vitro* assembled VLP nanoreactors with different NAD loading. Scale bar: 200 nm.

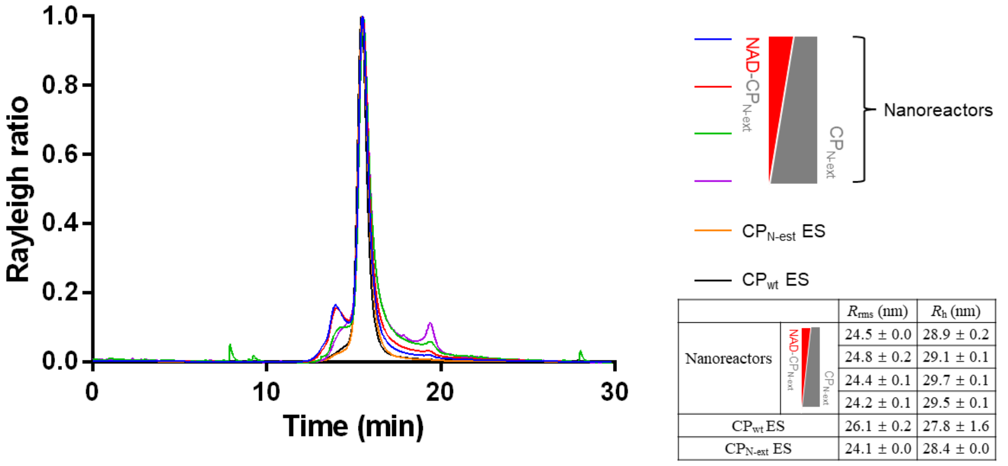

		R_{rms} (nm)	R_h (nm)
Nanoreactors	NAD-CPN-ext → CPN-ext	24.5 ± 0.0	28.9 ± 0.2
		24.8 ± 0.2	29.1 ± 0.1
		24.4 ± 0.1	29.7 ± 0.1
		24.2 ± 0.1	29.5 ± 0.1
CP_{wt} ES		26.1 ± 0.2	27.8 ± 1.6
$CP_{N\text{-}ext}$ ES		24.1 ± 0.0	28.4 ± 0.0

Supplementary Fig. 13. Characterization of *in vitro* assembled VLP nanoreactors with different NAD loading by SEC-QELS (mean ± s.e.m., n=3). Rayleigh ratio is displayed in relative scale.

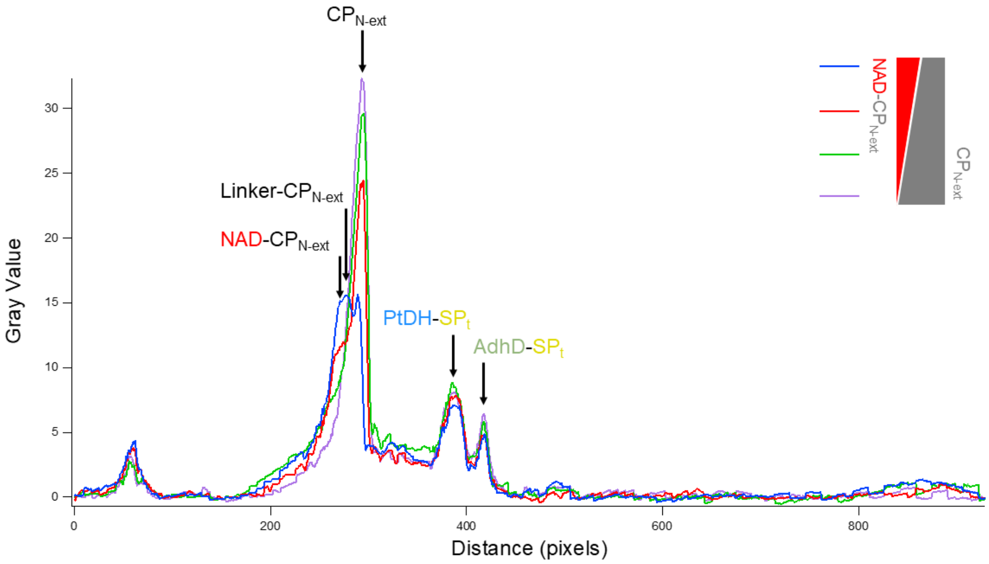

Supplementary Fig. 14. Densitometry plot of SDS-PAGE shown in Fig. 3aii.

Analysis: Each component of the nanoreactors is quantified by the peak area in the densitometry plot. This quantification is only an estimation, since the Gaussian fitting cannot be applied due to noisy baseline and the tailing of the protein bands.

	NAD-$CP_{N\text{-}ext}$ nanoreactors			$CP_{N\text{-}ext}$ nanoreactor
	NAD-$CP_{N\text{-}ext}$ → $CP_{N\text{-}ext}$			
NAD-CP	37%	19%	7%	-
linker-CP	10%	8%	10%	-
Unlabled CP	31%	47%	60%	74%
PtDH band	16%	20%	15%	18%
AdhD band	6%	6%	7%	8%
NAD-CP % (out of total CP)	48%	25%	9%	0%

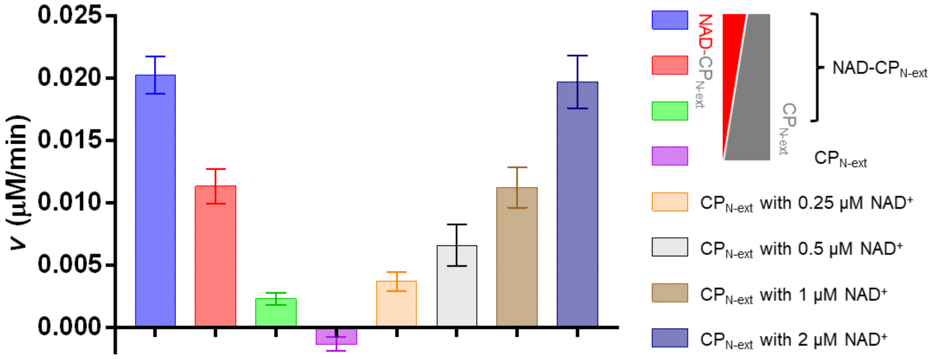

Supplementary Fig. 15. The steady state rates of *in vitro* assembled VLP nanoreactors with different NAD loading, determined by the average velocity between 90-180 min.

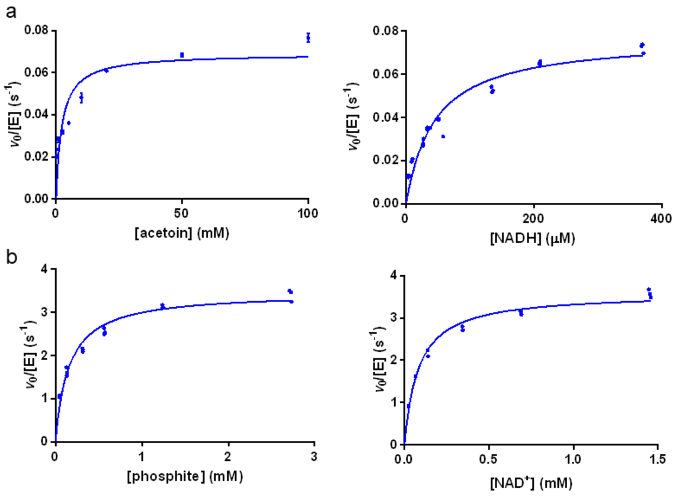

	k_{cat}	K_m
Acetoin	0.06896 ± 0.00341 s^{-1}	2.311 ± 0.504 mM
NADH	0.07702 ± 0.00327 s^{-1}	44.70 ± 6.02 µM

	k_{cat}	K_m
Phosphite	3.467 ± 0.096 s^{-1}	0.1574 ± 0.0176 mM
NAD+	3.590 ± 0.066 s^{-1}	82.27 ± 6.15 µM

Supplementary Fig. 16. Michaelis-Menten constants of AdhD (a) and PtDH (b) encapsulated in P22 VLPs (mean ± s.e.m, n = 3).

Analysis: The kinetic measurement was carried out at 20 mM acetoin and 0.4 mM sodium phosphite. Under this condition, the apparent turnover number of the enzymes can be calculated based on Michaelis-Menten equation:

$$k'_{\mathrm{cat,PtDH}} = \frac{k_{\mathrm{cat,PtDH}}[\mathrm{phosphite}]}{K_{\mathrm{m,acetoin,PtDH}}+[\mathrm{phosphite}]} = \frac{3.467\ \mathrm{s}^{-1}\times 0.4\ \mathrm{mM}}{0.1574\ \mathrm{mM}+0.4\ \mathrm{mM}} = 2.488\ \mathrm{s}^{-1};$$

$$k'_{\mathrm{cat,AdhD}} = \frac{k_{\mathrm{cat,AdhD}}[\mathrm{acetoin}]}{K_{\mathrm{m,acetoin,AdhD}}+[\mathrm{acetoin}]} = \frac{0.06896\ \mathrm{s}^{-1}\times 20\ \mathrm{mM}}{2.311\ \mathrm{mM}+20\ \mathrm{mM}} = 0.06182\ \mathrm{s}^{-1}.$$

The apparent turnover of PtDH is about 40 fold higher than that of AdhD.

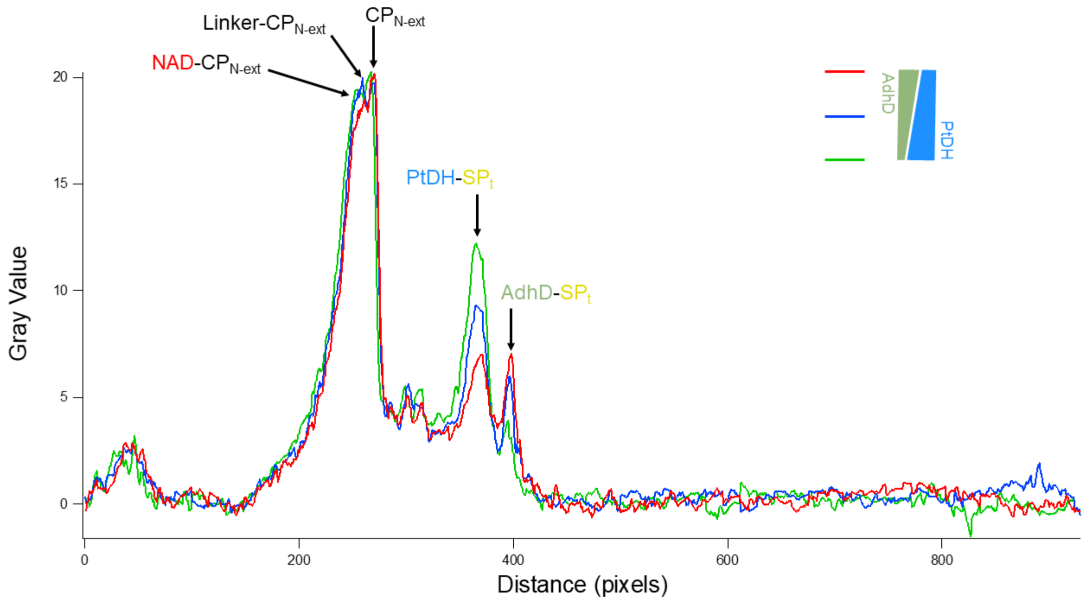

Supplementary Fig. 17. Densitometry plot of SDS-PAGE shown in Fig. 3bii.

Analysis: Each component of the nanoreactors is quantified by the peak area in the densitometry plot. This quantification is only an estimation, since the Gaussian fitting cannot be applied due to noisy baseline and the tailing of the protein bands.

	AdhD		PtDH
NAD-CP	35%	33%	33%
Linker-CP	11%	14%	11%
Unlabeled CP	31%	28%	28%
PtDH	14%	19%	25%
AdhD	8%	6%	3%
PtDH:AdhD	1.7	3.0	7.0

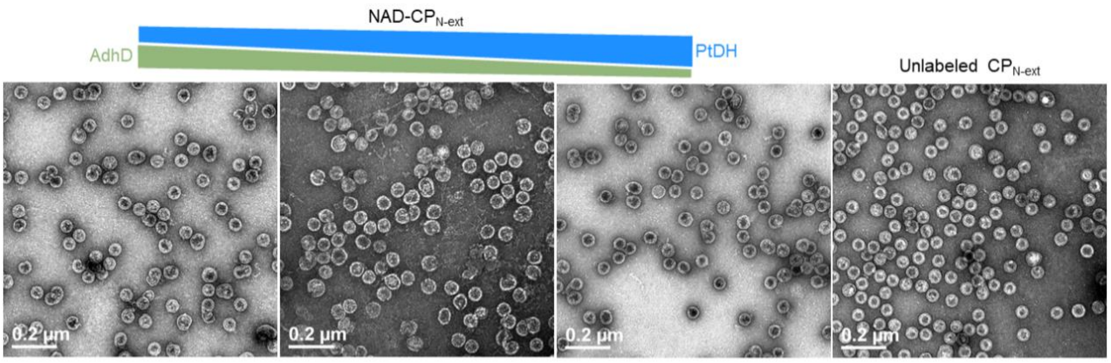

Supplementary Fig. 18. TEM images of *in vitro* assembled particles with different enzyme stoichiometry. Scale bar: 0.2 µm.

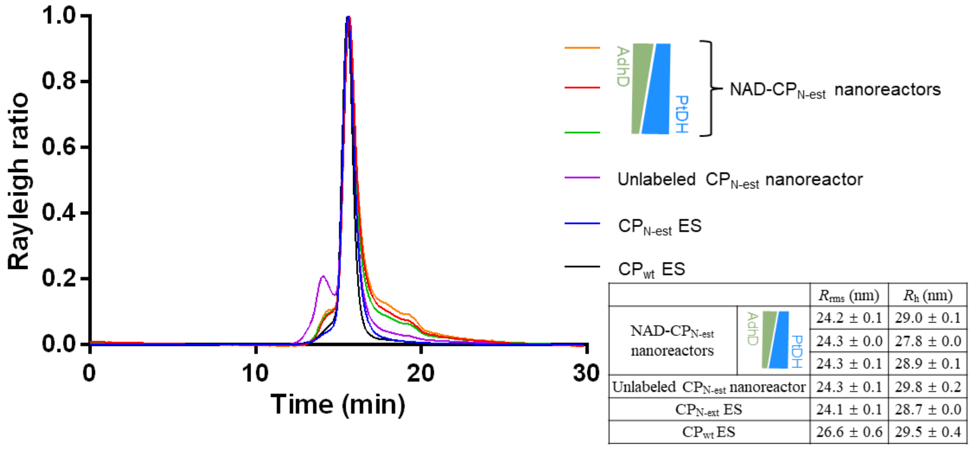

Supplementary Fig. 19. Characterization of *in vitro* assembled particles with different enzyme stoichiometry by SEC-QELS (mean ± s.e.m., n=3). Rayleigh ratio is displayed in relative scale.

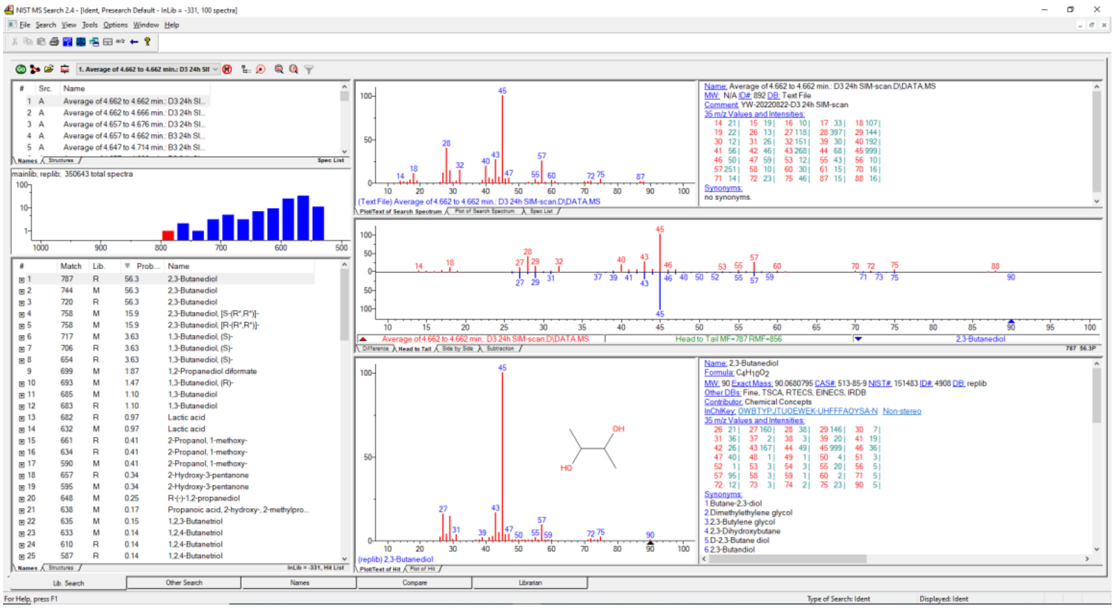

Supplementary Fig. 20. NIST MS search result on GC-MS total ion chromatogram (TIC) at 4.662 min. The result confirms presence of 2,3-butanediol after the acetoin reduction reaction and its elution time is 4.662 min.

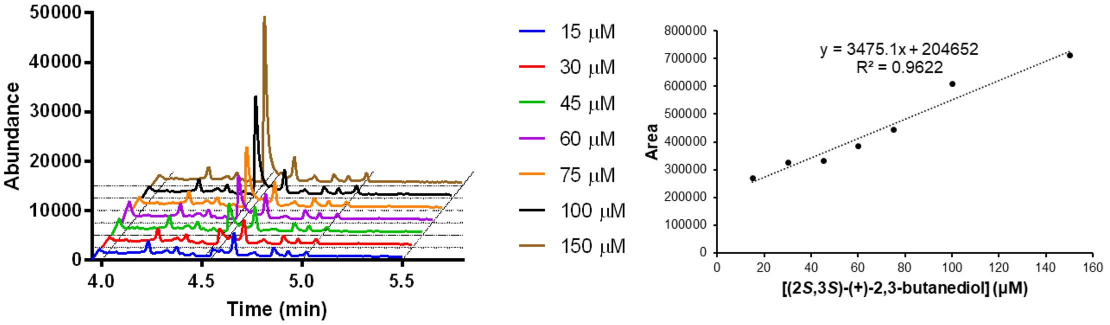

Supplementary Fig. 21. GC-MS selected ion monitoring (SIM) chromatograms of (2*S*,3*S*)-(+)-2,3-butanediol standard at different concentrations (left). A calibration curve is made peak area and concentration of (2*S*,3*S*)-(+)-2,3-butanediol (right).

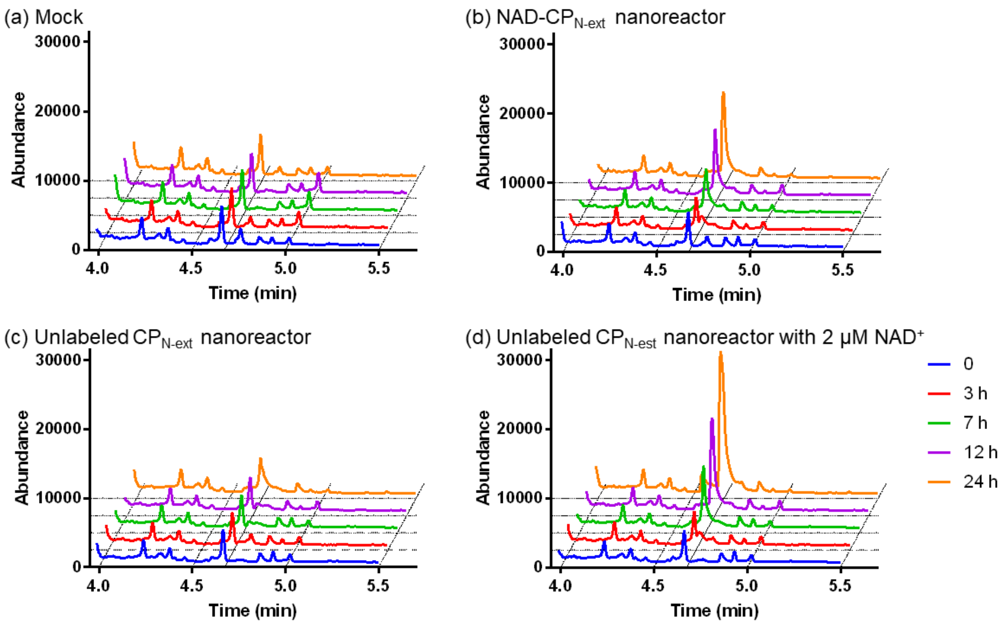

Supplementary Fig. 22. GC-MS selected ion monitoring (SIM) chromatograms of the acetoin reduction reaction at different time points. (a) Mock. (b) NAD-$CP_{N\text{-ext}}$ particles. (c) Unlabeled $CP_{N\text{-ext}}$ particles. (d) Unlabeled $CP_{N\text{-ext}}$ particles supplemented with 2 µM NAD^+.

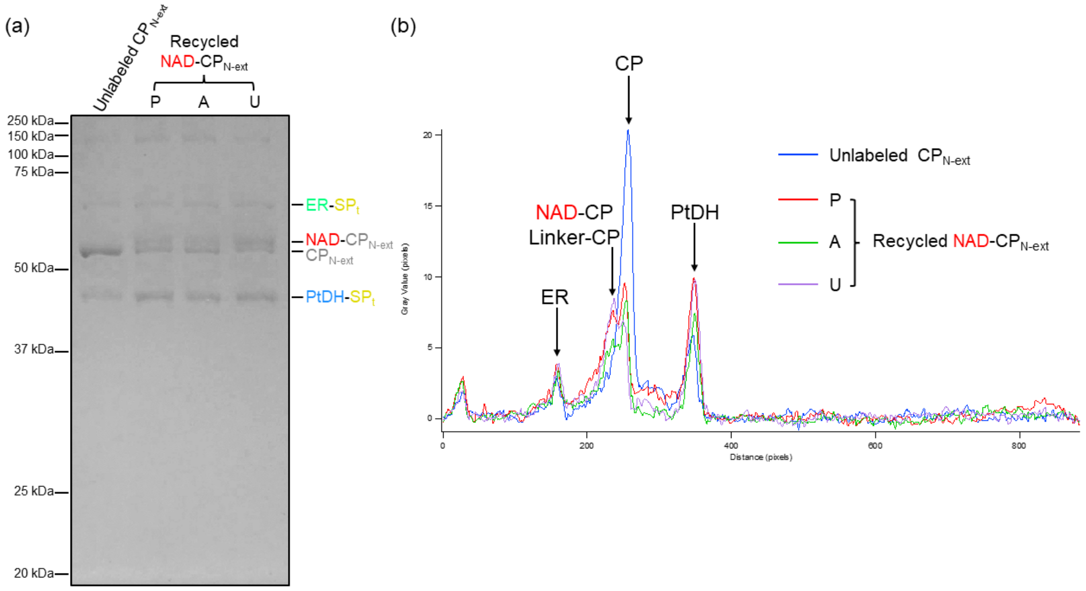

Supplementary Fig. 23. (a) SDS-PAGE of nanoreactors assembled from PtDH, ER, and recycled NAD-CP$_{N\text{-}ext}$. (b) Densitometry analysis of the SDS-PAGE. P = NAD-CP$_{N\text{-}ext}$ recycled from *in vitro* assembled particles. A = NAD-CP$_{N\text{-}ext}$ recycled from *in vitro* assembly aggregates. U = NAD-CP$_{N\text{-}ext}$ recycled from unassembled protein of assembly.

Analysis: Each component of the nanoreactors is quantified by the peak area in the densitometry plot. This quantification is only an estimation, since the Gaussian fitting cannot be applied due to noisy baseline and the tailing of the protein bands. NAD-CP and linker-CP are not resolved well in this gel.

Component	CP$_{N\text{-}ext}$	Recycled NAD-CP$_{N\text{-}ext}$		
		P	A	U
NAD-CP and Linker-CP	-	33%	31%	36%
Unlabled CP	55%	27%	24%	20%
ER	17%	12%	18%	15%
PtDH	27%	27%	26%	29%

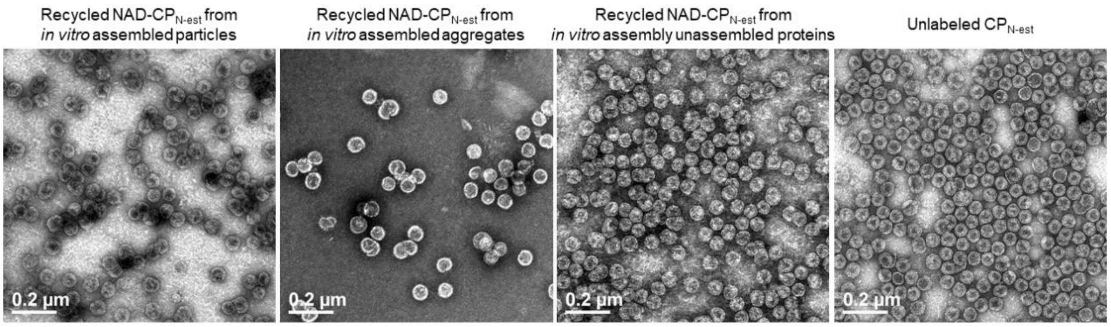

Supplementary Fig. 24. TEM images of nanoreactors assembled from PtDH, ER, and recycled NAD-CP$_{N\text{-}ext}$. Scale bar: 0.2 µm.

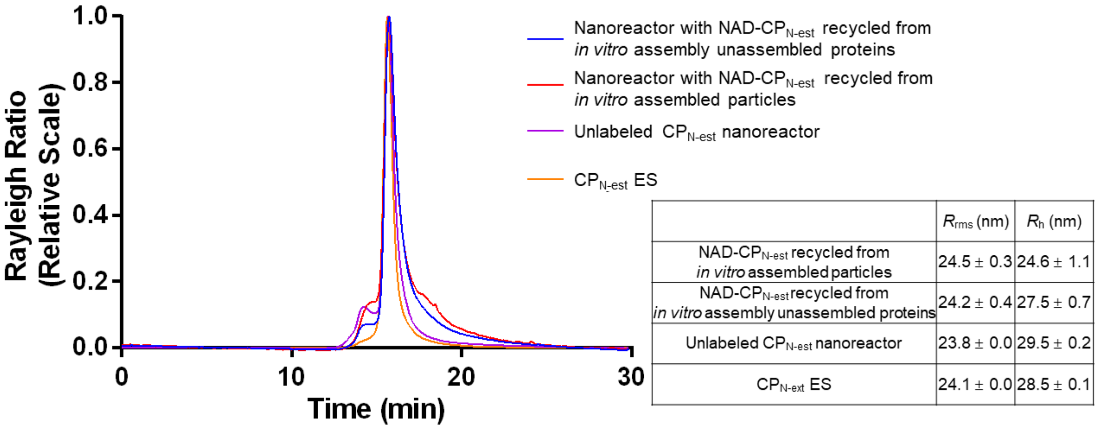

	R_{rms} (nm)	R_h (nm)
NAD-CP$_{N\text{-}est}$ recycled from *in vitro* assembled particles	24.5 ± 0.3	24.6 ± 1.1
NAD-CP$_{N\text{-}est}$ recycled from *in vitro* assembly unassembled proteins	24.2 ± 0.4	27.5 ± 0.7
Unlabeled CP$_{N\text{-}est}$ nanoreactor	23.8 ± 0.0	29.5 ± 0.2
CP$_{N\text{-}ext}$ ES	24.1 ± 0.0	28.5 ± 0.1

Supplementary Fig. 25. SEC-QELS results of nanoreactors assembled from PtDH, ER, and recycled NAD-CP$_{N\text{-}ext}$ (mean ± s.e.m., n=3).

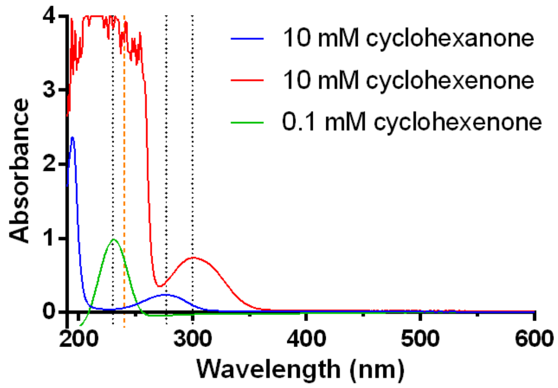

	A_{230}	A_{277}	A_{300}	A_{240}
10 mM cyclohexanone	0.039076	0.23267	0.09256	0.056609
10 mM cyclohexenone	-	0.39758	0.71983	-
0.1 mM cyclohexenone	0.97152	-0.050662	-0.037061	0.70195

Supplementary Fig. 26. UV-vis spectra of cyclohexanone and cyclohexenone. Cyclohexanone has maximum absorbance at 277 nm. Cyclohexenone has maximum absorbance at 230 nm and 240 nm. The concentration of cyclohexenone used in the kinetic study was 150 µM. Due to strong absorbance of P22 VLP nanoreactors at 230 nm, A_{240} was monitored to detect the consumption of cyclohexenone.

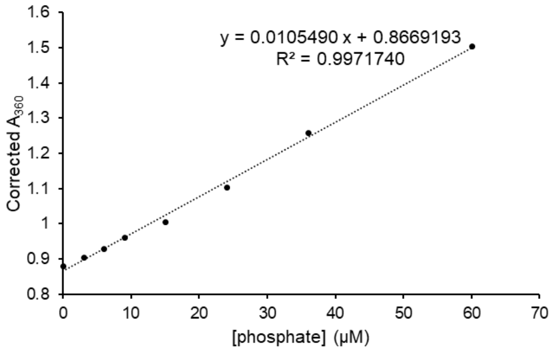

Supplementary Fig. 27. Calibration curve of EnzChek phosphate assay. The difference in extinction coefficient between the substrate (2-amino-6-mercapto-7-methylpurine riboside, MESG) and the product (ribose 1-phosphate and 2-amino-6-mercapto-7-methylpurine) of EnzChek phosphate assay in 100 mM HEPES (pH 7.2) is thus calculated as 10549.0 M^{-1} cm^{-1}.

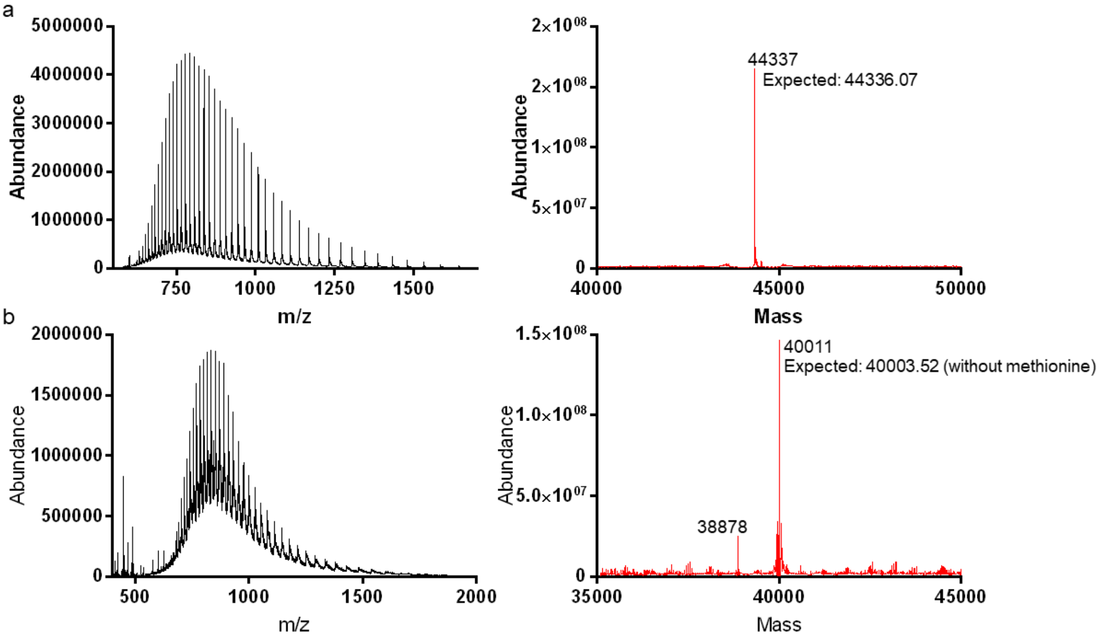

Supplementary Fig. 28. LC-MS of PtDH-SP_t (a) and AdhD-SP_t (b). A small fraction of AdhD-SP_t was shown as a cleavage product (loss of the last 10 amino acid residues; expected: 38867.03 Da).

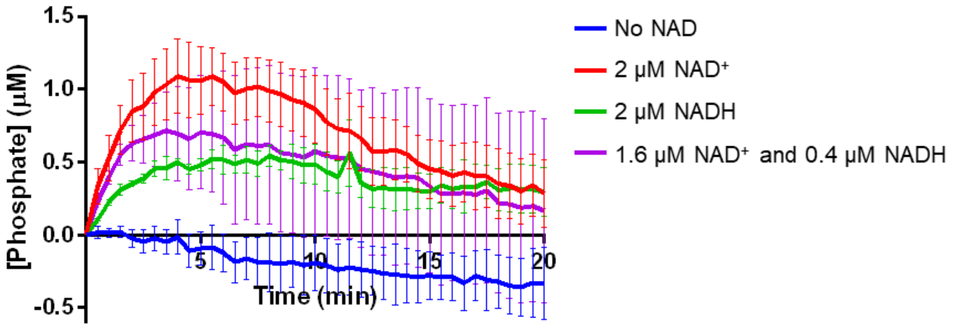

Supplementary Fig. 29. Burst phase is diminished when the percentage of reduced form of NAD increases. A group of 1.6 μM NAD^+ and 0.4 μM NADH is included, which is similar to the NAD^+:NADH ratio in the NAD-maleimide molecule before bioconjugation (20% NAD in reduced form, supplementary Fig. 2).

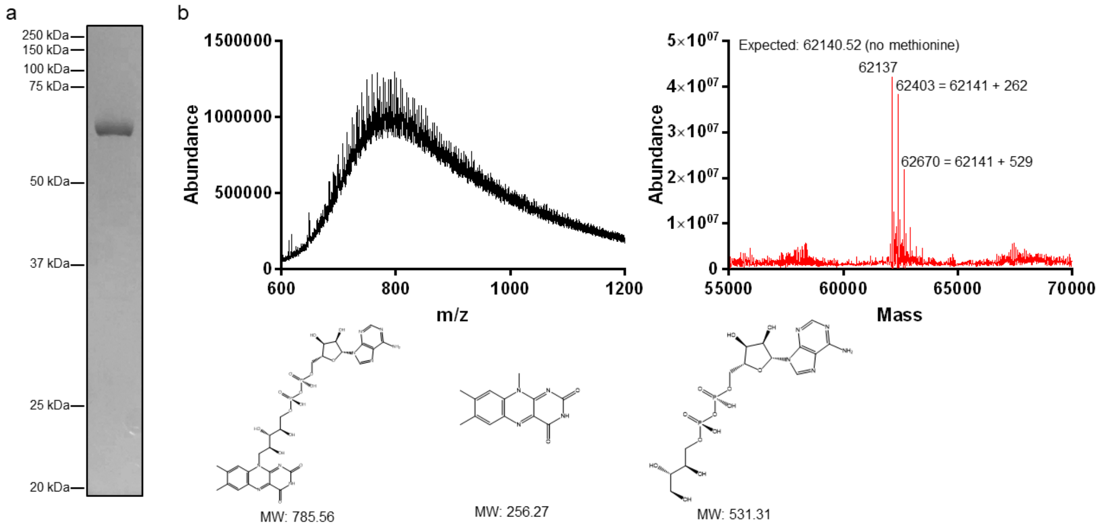

Supplementary Fig. 30. Characterization of ER-SP_t by SDS-PAGE (a) and LC-MS (b). The observed molecular masses higher than expected might be due to binding with cleaved FAD, a prosthetic group of ER.[1]

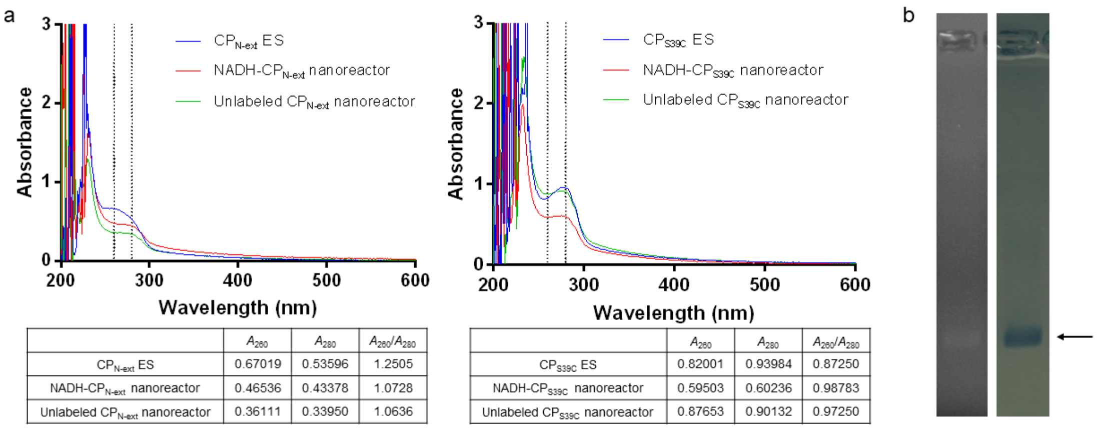

	A_{260}	A_{280}	A_{260}/A_{280}
$CP_{N\text{-}ext}$ ES	0.67019	0.53596	1.2505
NADH-$CP_{N\text{-}ext}$ nanoreactor	0.46536	0.43378	1.0728
Unlabeled $CP_{N\text{-}ext}$ nanoreactor	0.36111	0.33950	1.0636

	A_{260}	A_{280}	A_{260}/A_{280}
CP_{S39C} ES	0.82001	0.93984	0.87250
NADH-CP_{S39C} nanoreactor	0.59503	0.60236	0.98783
Unlabeled CP_{S39C} nanoreactor	0.87653	0.90132	0.97250

Supplementary Fig. 31. Nucleic acid contamination in $CP_{N\text{-}ext}$ ES. (a) UV-vis spectra of ES and nanoreators. The A_{260}/A_{280} ratio of $CP_{N\text{-}ext}$ indicates nucleic acid contamination. The decreased A_{260}/A_{280} ratio of $CP_{N\text{-}ext}$ nanoreactors indicates less nucleic acid contamination. (b) Native agarose gel analysis of $CP_{N\text{-}ext}$ ES confirms nucleic acid binds to the VLPs. Left: ethidium bromide staining. Right: Coomassie staining.

Total NAD^+ added (c_{NAD}, μM)	[NADH] at steady state (μM)	[NAD^+] at steady state (μM)	NADH:NAD^+ ratio
0.25	0.2444	0.0056	43.6
0.5	0.4889	0.0111	44.0
1	0.9781	0.0219	44.7
2	1.9571	0.0429	45.6

Supplementary Table 1. The concentrations of NADH and NAD^+, and their ratio required in the steady state of PtDH-AdhD coupled reaction (see calculation below). These concentrations should remain constant when the steady state is maintained.

Analysis:

Based on Michaelis-Menten equation, the rate of each step of the PtDH-AdhD coupled can be calculated:

$$V_{AdhD} = \frac{k'_{\mathrm{cat,AdhD}}[\mathrm{NADH}]c_{\mathrm{AdhD}}}{K_{\mathrm{m,NADH,AdhD}} + [\mathrm{NADH}]} \quad (1), \text{ and}$$

$$V_{PtDH} = \frac{k'_{\mathrm{cat,PtDH}}[\mathrm{NAD^+}]c_{\mathrm{PtDH}}}{K_{\mathrm{m},NAD+,\mathrm{PtDH}} + [\mathrm{NAD^+}]} \quad (2),$$

where $k'_{\mathrm{cat,AdhD}}$ (0.06182 s^{-1}) and $k'_{\mathrm{cat,PtDH}}$ (2.488 s^{-1}) are apparent turnover rate under the substrate condition used (see supplementary Fig. 16), $K_{\mathrm{m,NADH,AdhD}}$ (44.70 μM) and $K_{\mathrm{m},NAD+,\mathrm{AdhD}}$ (82.27 μM) are Michaelis constants (see supplementary Fig. 16), c_{AdhD} and c_{PtDH} are total concentrations of the enzymes. Based on the densitometry analysis (supplementary Fig. 9), the PtDH amount is roughly 2 times of AdhD amount:

$$c_{\mathrm{PtDH}} = 2c_{\mathrm{AdhD}} \quad (3).$$

The total NAD concentration is constant in the solution:

$$c_{\mathrm{NAD}} = [\mathrm{NADH}] + [\mathrm{NAD^+}] \quad (4).$$

When the PtDH-AdhD coupled reaction reaches the steady state, the velocities of the two steps are equal:

$$V_{\mathrm{AdhD}} = V_{\mathrm{PtDH}} \quad (5).$$

Apply equations (1) to (4) to equation (5), [NADH] can be solved:

$$[\mathrm{NADH}] = \frac{-\alpha + \sqrt{\alpha^2 + 8k'_{\mathrm{cat,PtDH}}c_{\mathrm{NAD}}K_{\mathrm{m,NADH,AdhD}}(2k'_{\mathrm{cat,PtDH}} - k'_{\mathrm{cat,AdhD}})}}{4k'_{\mathrm{cat,PtDH}} - 2k'_{\mathrm{cat,AdhD}}} \quad (6), \text{ where}$$

$$\alpha = k'_{\mathrm{cat,AdhD}}\left(K_{\mathrm{m},NAD+,\mathrm{PtDH}} + c_{\mathrm{NAD}}\right) - 2k'_{\mathrm{cat,PtDH}}\left(c_{\mathrm{NAD}} - K_{\mathrm{m,NADH,AdhD}}\right) \quad (7).$$

The equation (6) is used to calculate the concentration of NADH at the steady state.

The phosphate production reflects the rate of PtDH, and consequently NADH production as well. Clearly, at the end of the burst phase, the amount of PtDH was produced (Fig. 2c) is much lower than the concentration of NADH required in the steady state. This suggests that, at the end of the burst phase, the system did not reach steady state yet and was still far away from the steady state.

Number of molecules retained inside P22 VLP capsid (N)	Theoretical local concentration
420	15.0 mM
300	10.7 mM
200	7.15 mM
150	5.35 mM
100	3.57 mM
50	1.79 mM
25	890 uM
10	357 uM
5	179 uM
1	35.7 uM

Supplementary Table 2. Theoretical local concentration of molecules that are retained inside the P22 VLP capsid. The concentration was calculated by

$$\text{local concentration} = (N/N_A)/V_{cavity},$$

where N is the number of molecules retained inside P22 VLP capsid, N_A is Avogadro constant, and V_{cavity} is the volume of P22 capsid cavity (46450 nm^3).

Note: This calculation assumes the molecules are completely retained and can freely diffuse inside the capsid cavity, and does not reflect the situation of the immobilized NAD. The encapsulated enzymes may not have full access to the immobilized NAD.

Methods

No unexpected or unusually high safety hazards were encountered.

Recombinant protein expression

To recombinantly express the enzymes (PtDH-SP_t AdhD-SP_t, or ER-SP_t), a pBAD-based plasmid harboring the enzyme gene was transformed in E. cloni EXPRESS Competent Cells (Biosearch Technologies). To recombinantly produce P22 VLPs, a pRSF-based plasmid containing CP gene (CP_{S39C} or $CP_{N\text{-}ext}$) and a pBAD-based plasmid gene containing SP_t gene were co-transformed in E. cloni EXPRESS Competent Cells. The cells were grown in 1 L LB medium supplemented with antibiotics (100 mg/L ampicillin for enzymes; 100 mg/L ampicillin and 30 mg/L kanamycin for P22 VLPs) at 37°C, 220 rpm until OD_{600} reached about 0.5. Inducers were then added (1 g/L L-arabinose for enzymes; 1 g/L L-arabinose and 0.3 mM isopropyl β-thiogalactopyranoside for P22 VLPs), followed by incubation (37°C, 220 rpm, 4 h for enzymes; 30°C, 220 rpm, overnight for P22 VLPs). The cells were then harvested by centrifugation at 4500 rpm, 15 min, 4°C, and then stored at -80°C.

Recombinant protein purification

The cells were thawed from -80°C, and resuspended in the lysis buffer (50 mM sodium phosphate, 100 mM NaCl; pH 7.8 for enzymes; pH 7.0 supplemented with 2 mM EDTA for P22 VLPs). Lysozyme (1.5 mg), DNase (2 mg), and RNase (3 mg) were added to the cells, followed by incubation at room temperature (r.t.) for 30 min and then sonicated for 2 min at 50% amplitude twice. The cell lysate was obtained by collecting the supernatant after centrifugation at 12000 rpm, 4°C for 45 min.

To purify the enzymes, the cell lysate was filtered through a 0.45 μm syringe filter and applied to a HisTrap HP column (Cytiva) using a Bio-rad NGC FPLC. The enzyme was purified and eluted with an imidazole gradient (0-500 mM in lysis buffer), and then dialyzed into the assembly buffer (50 mM Tris, 25 mM sodium chloride, 2 mM EDTA, 3 mM 2-mercaptoethanol, 1% glycerol, pH 7.6). The purified enzymes were stored at 4°C.

To purify P22 VLPs, the cell lysate was layered onto a 35% (w/v) sucrose cushion and ultracentrifuged using Thermo Scientific Sorvall WX Ultra Series Centrifuge at 45000 rpm, 50 min, 4°C (rotor: F50L-8x39). The pellet was resuspended in lysis buffer, and then purified through a Sephacryl S-500 column (GE Healthcare Life Sciences) using a Bio-rad Biologic Duoflow FPLC. To obtain the empty shell (ES) form of the VLPs, SP was removed by treating the purified VLPs with 0.5 M GdnHCl in lysis buffer for 1 h and ultracentrifuged (45000 rpm, 50 min, 4°C) to pellet the VLP. Then the pellet was dissolved in the lysis buffer. This process was usually repeated at least three times in order to remove all SP. The VLP ES was stored at 4°C.

Synthesis of NAD-Maleimide

The synthetic route (supplementary Fig. 1) and methods for NAD-maleimide synthesis were adapted from a previously reported method.[2]

To synthesize 8-Br-NAD^+, 1 g β-nicotinamide adenine dinucleotide (Chem-Impex, Cat# 00229) was dissolved in 15 mL 500 mM sodium acetate buffer (pH 4.5) in a 100-mL flask. 0.4 mL Br_2 was added dropwise with stirring at the highest speed. Then the system was then kept with stirring for ~2 hrs at room temperature (r.t.). After the reaction completed, the UV-vis spectrum of the product was measured, and peak absorbance had shifted from 259 nm (NAD^+) to 264 nm. Then excess Br_2

was extracted using chloroform until the organic phase was clear. The aqueous layer was then recovered, and dialyzed through 0.5-1k MWCO membrane into 2 L water overnight, twice. The product was then lyophilized and stored at -80°C.

To synthesize 8-Br-NADH, 20 mL 1.3% sodium bicarbonate was added to a two-neck round-bottom flask, which was then bubbled with argon for at least 1 hr. Then 300 mg 8-NAD^+-Br was added to the flask through side neck, followed by addition of 150 mg sodium dithionite (Fisher). The system was then covered with aluminum foil and stirred for ~4 hrs at r.t. while bubbling argon. UV-vis spectrum was measured, and the completion of the reaction was indicated by A_{265}:A_{340} ratio close to about 3. The reaction was stopped by being mixing with 200 mL cold acetone, and the product was precipitated and subsequently recovered by collecting the pellet after centrifugation at 12000 rpm, 4°C for 2 min. The product was stored at -80 °C.

To synthesize 8-NH_2-NADH, all the product of the previous step was thawed and mixed with 10 mL DMSO in a two-neck round-bottom flask. Stirring dissolved 8-Br-NADH in DMSO while impurities including salt stayed undissolved. The system was blanketed with argon, placed under a reflux condenser, and covered with aluminum foil. The flask was then heated to 60 °C with stirring. 1.4 ml ethylenedioxy)bis(ethylamine) (Sigma) was dissolved in 10 mL DMSO, which was then added to the flask from the side neck. The system was then stirred for 6 hrs, and subsequently cooled to r.t. overnight. The mixture was then centrifuged at 12000 rpm, 4 °C for 2 min, and the supernatant was collected. The product was precipitated by adding the supernatant to 200 ml cold acetone, and subsequently recovered by collecting the pellet after a second centrifugation. Then the product was dissolved in 10 mL DMSO, and precipitated again using 150 ml cold acetone. Finally, the product was recovered by collecting the pellet after a third centrifugation, and stored at -80 °C.

To synthesize NAD-maleimide, 325 mg N-(3-Dimethylaminopropyl)-N′-ethylcarbodiimide hydrochloride (Sigma-Aldrich) and 60 mg N-hydroxysuccinimide (Aldrich) was dissolved in 5 mL DMSO in a glass flask, followed by addition of 30 μL triethanolamine. 100 mg MAL-dPEG®$_8$-acid (Quanta Biodesign) was added to the mixture, and stirred for 1 hr at r.t. at dark. Then about a quarter of the product from the previous step (synthesis of 8-NH_2-NADH) was added to the reaction, followed by stirring in the dark for 8 hrs at r.t.. The crude product was precipitated by adding the reaction mixture to 80 ml cold acetone, and recovered by collecting the pellet after centrifugation at 12000 rpm, 4 °C for 2 min. The crude product was stored in -80 °C temporarily. To purify NAD-maleimide from the crude product, the product was thawed from -80 °C and dissolved in ~500 μL 50% methanol (v/v in water), and then applied to a BioRad disposable chromatography 20 mL column (part # 9704652) packed with 17-18 mL Cosmosil 75C18-OPN resin (Nacalai). The mobile phase used in this gravity chromatography was 50% methanol (v/v in water), and UV-vis spectroscopy was used to monitor the elution profile. The purified product was then lyophilized and stored -80 °C (average yield: 23% from the starting unmodified NAD^+).

NAD-CP bioconjugation

To reduce the addressable cysteine on $CP_{N\text{-}est}$ and CP_{S39C}, P22 VLP ES was diluted to 1-2 mg/ml in lysis buffer (50 mM sodium phosphate, 100 sodium chloride, 2 mM EDTA, pH 7.0), and incubated with 2 mM dithiothreitol (DTT) at 4 °C overnight. Then additional 2 mM DTT was added, and the protein was incubated at 45 °C for 1 h. To remove the reducing agents the P22 VLP sample was ultracentrifuged (45000 rpm, 50 min, 4°C), and the pellet was resuspended in the lysis buffer. This was repeated twice to completely remove DTT.

To label CP with NAD, NAD-maleimide was added to the reduced P22 VLP ES, with a CP:NADH molar ratio of 1:2. Here, CP concentration was determined using Bradford assay, while NADH concentration was determined by A_{340}. Because only about 20% of NAD-maleimide is in the reduced form (supplementary Fig. 2), the actual molar ratio of CP:NAD-maleimide was about 1:10. The bioconjugation was done by incubation at room temperature (r.t.), in the dark overnight. The reaction was quenched by adding 0.1% 2-mercaptoethanol. The P22 VLP sample was ultracentrifuged (45000 rpm, 50 min, 4°C), and the pellet was resuspended in assembly buffer (50 mM Tris, 25 mM sodium chloride, 2 mM EDTA, 3 mM 2-mercaptoethanol, 1% glycerol, pH 7.6) for the subsequent *in vitro* assembly.

In vitro assembly

1 mL of P22 VLP ES with a CP concentration of 80 μM (determined by Bradford assay) was mixed, in an 1:1 volume ratio, with 6 M guanidine hydrochloride (GdnHCl) in the assembly buffer. This resulted in denatured free CP in solution for *in vitro* assembly (40 μM CP, 3 M GdnHCl, in the assembly buffer).

The concentration of the enzymes in the assembly buffer was quantified by A_{280} (extinction coefficient: 27960 M^{-1} cm^{-1} for PtDH-SP_t, 53860 M^{-1} cm^{-1} for AdhD-SP_t, and 37360 M^{-1} cm^{-1} for ER-SP_t). To make enzyme solution for *in vitro* assembly, the enzymes were mixed with total enzyme concentration of 40 μM, where the enzyme ratio can be adjusted here to vary the enzyme stoichiometry in the assembled particles.

To start *in vitro* assembly, the enzyme solution was added to the denatured CP solution (1:1 volume ratio) at r.t., resulting in a protein mixture solution containing 20 μM CP, 20 μM enzyme, and 1.5 M GdnHCl. The assembly was initiated by immediate dialysis of the mixture solution into fresh assembly buffer twice for a total of 15-18 h at r.t., using 6-8k MWCO dialysis membrane. Usually, 4 mL mixture solution was dialyzed into 500 mL assembly buffer each time, and the volume of assembly buffer was proportionally adjusted based on the volume of the protein mixture solution.

After dialysis, the solution was centrifuged at 17000 g, 2 min, r.t., to remove assembly aggregates. The assembled particles were then pelleted twice by ultracentrifugation at 43000 rpm, 40 min, 4°C (rotor: F50L-24x1.5) using Thermo Scientific Sorvall WX Ultra Series Centrifuge, followed by resuspension in buffer (100 mM HEPES, pH 7.2). The unassembled proteins were mostly separated into the supernatant of the first ultracentrifugation.

Bradford Assay

The assay was carried by mixing 10 μL protein with 200 μL Coomassie Protein Assay Reagent (Thermo) in a 96-well plate and incubating for 10 min at r.t., followed by reading OD_{595} using BioTek Cytation5 plate reader. The protein standard used in Bradford assay is CP_{S39C} P22 VLP ES, whose concentration was determined by A_{280} (extinction coefficient: 45045 M^{-1} cm^{-1}) in denaturing condition (4.5 M GdnHCl). The calibration curve shows a linear response in OD_{595} within the range of 0-21 μM CP_{S39C}.

Monitoring phosphate production in PtDH-AdhD coupled reaction

Phosphate production was monitored using EnzChek Phosphate Assay Kit (Invitrogen). Basically, the reaction was carried out at 37 °C in the reaction buffer (100 mM HEPES, pH 7.2) at 120 μL scale in 96-well half-area plates. Each reaction contains 1.8 μM P22 VLP nanoreactors

(concentration determined using Bradford assay), 20 mM acetoin (Acros), 400 μM sodium phosphite (Acros), 200 μM 2-amino-6-mercapto-7-methylpurine riboside (MESG; assay substrate), and 1 U/mL purine nucleoside phosphorylase (PNP), where the concentrations of MESG and PNP are recommended by the manufacturer. Unmodified NAD^+ was added when noted. A mock reaction was included without P22 VLP nanoreactors to obtain the background rate. The kinetics of the reactions were monitored by reading A_{360} using BioTek Cytation5 plate reader under pathlength correction mode, and the data were normalized by correction from A_{360} at time zero and the rate of mock reaction, and the difference in extinction coefficient between MESG and its product (ε, supplementary Fig. 27):

$$[\text{phosphate}] = \frac{(A_{360,\text{t}} - A_{360,t=0}) - (A_{360,\text{mock,t}} - A_{360,\text{mock},t=0})}{\varepsilon}.$$

Monitoring acetoin production in PtDH-AdhD coupled reaction

The reaction was carried out at 37 °C in the reaction buffer (100 mM HEPES, pH 7.2). Each reaction contains 1.8 μM P22 VLP nanoreactors (concentration determined using Bradford assay), 20 mM acetoin, and 400 μM sodium phosphite. Unmodified NAD^+ was added when noted. A mock reaction was included without P22 VLP nanoreactors. The reaction was initiated in a total volume of 525 uL, and then immediately aliquoted into 105 μL in PCR tubes, which were incubated at 37 °C on a Thermo Scientific Arktik thermal cycler. At each time point, 100 μL reaction was taken from one aliquot and quenched by adding 10 μL hydrochloric acid (1 N), followed by storage at -80 °C.

To prepare the samples for analysis, the samples were thawed and neutralize by adding 10 uL sodium hydroxide (1N). 120 μL ethyl acetate was then added to extract 2,3-butanediol. To increase the extraction efficiency, 0.1 g NaCl was added to saturate the aqueous phase followed by mixing (vortexing). After centrifugation at 17000 g, 2 min, r.t., the ethyl acetate phase was taken for GC-MS analysis.

The samples were analyzed by Agilent 6890N Gas Chromatograph (GC) coupled with an Agilent 5973 Inert Mass Selective Detector (MSD). 1 μL of the sample was injected with a split ratio of 2:1 into an Agilent DB-5MS column (30m, 0.25mm, 0.25μm), with a total inlet flow of helium at 6 mL/min. After sample injection, the oven temperature was held initially 40 °C for 3 min, and then ramped to 280 °C at 20 °C/min and held there for 2-min. The detector was on only between elution time 4 min and 5.5 min, and in the acquisition mode of selected ion monitoring (SIM)/scan (SIM: m/z 45, 57, 75, 90; scan: m/z range 10-100). 2,3-butanediol was confirmed by the total ion (scan) chromatograms using NIST MS Search 2.4 software, and quantified by integrating the peak area of 2,3-butanediol in the SIM chromatograms using OpenChrome (1.5.0.202209020347) software.

Recycling of NAD-$CP_{N\text{-ext}}$

To recycle NAD-$CP_{N\text{-ext}}$ from *in vitro* assembled particles and aggregates from *in vitro* assembly, the particles were disassembled, and the aggregates were dissolved, in 3 M GdnHCl (in the assembly buffer). Then the proteins in this denaturing condition was supplemented with 5 mM imidazole, and then incubated with cOmplete His-tag purification resin (Roche, about 30 mg total protein per mL of the resin) at r.t. for 1 h. The mixture was then centrifuged at 4500 rpm, 5 min, r.t. and both the supernatant and resin (pellet) were collected for analysis. The recycled NAD-$CP_{N\text{-ext}}$ was in the supernatant, which was then diluted with 3 M GdnHCl (in the assembly buffer) to 40 μM, serving

as the CP solution for further *in vitro* assembly.

To recycle NAD-$CP_{N\text{-ext}}$ from unassembled proteins of *in vitro* assembly, the proteins were supplemented with 5 mM imidazole and then incubated with cOmplete His-tag purification resin with the same condition mentioned above but in native condition (assembly buffer). After centrifugation, the supernatant was concentrated using a MWCO 30k Amicon spin concentrator, and then used for *in vitro* assembly as an equivalent of P22 VLP ES mentioned in the *in vitro* assembly section.

Monitoring cyclohexenone consumption in PtDH-ER coupled reaction

The reaction was carried out at r.t. in phosphate buffer (50 mM sodium phosphate, 100 mM sodium chloride, pH 7.0) at 100 µL scale in quartz cuvettes. Each reaction contains 3 µM P22 VLP nanoreactors (concentration determined by Bradford assay), 150 µM 2-cyclohexen-1-one (Aldrich), and 5 mM sodium phosphite. Unmodified NAD^+ was added when noted. A mock reaction was included without P22 VLP nanoreactors to obtain the background rate. The kinetics of the reactions were monitored by measuring the absorbance (A_{240}) using an Agilent Cary 8454 UV−vis spectrophotometer, and the data were normalized by correction from A_{240} at time zero and the rate of mock reaction,

$$\text{Corrected } \Delta A_{240} = \Delta A_{240} - \Delta A_{240,\text{mock}} = (A_{240,t=0} - A_{240,\text{t}}) - (A_{240,\text{mock},t=0} - A_{240,\text{mock,t}}).$$

SDS-PAGE and densitometry analysis

The protein samples were analyzed by 12% sodium dodecyl sulfate–polyacrylamide gel electrophoresis (SDS-PAGE), which was stained by InstantBlue Coomassie Protein Stain (Abcam). When needed, the densitometry data were obtained by analyzing the gel picture in ImageJ (1.51j8). The data were then normalized and plotted in Igor Pro (6.37), and the peak area of the peaks were calculated when necessary.

TEM

P22 VLPs were diluted to A_{280} ~0.4 using water, and then 4.5 µL was applied to 400 mesh carbon-coated copper grids. After incubation for 1 min, excess sample was wicked away with filter paper. The grid was then stained with 4.5 µL 2% uranyl acetate for 15 sec, and then the stain was wicked away with filter paper. The images were taken on JEOL JEM-1010 transmission electron microscope.

SEC-QELS

P22 VLPs were centrifuged at 17000 g, 10 min, room temperature, and the supernatant was used for analysis. 25 µL P22 VLPs with A_{280} at 1.5-2.0 was injected onto a size-exclusion column (WTC-200S 5 µm, 2000 Å, 7.8 mm × 300 mm, Wyatt Technologies) utilizing an Agilent 1200 HPLC system, with a mobile phase of 50 mM sodium phosphate, 100 mM sodium chloride, 200 ppm sodium azide, pH 7.2 at 0.7 mL/min. The data were obtained by a Wyatt HELEOS multi-angle and quasi-elastic light scattering (MALS/QELS) detector and an Optilab rEX differential refractometer, and processed in Astra (5.3.14) software.

LC-MS

The proteins were diluted to ~1 mg/mL using the assembly buffer. The samples were analyzed

using a Waters UPLC-Synapt G2 HDMS system. The data were analyzed using MassLynx (V4.1 SCN916) software.

Protein Sequences

CP$_{N-ext}$

MGSCGSGGSSSSGGSSSSTRLSERLTLKPRGKQISSAPHADQGGSGGSSSSGGSSSSALNEG
QIVTLAVDEIIETISAITPMAQKAKKYTPPAASMQRSSNTIWMPVEQESPTQEGWDLTDKA
TGLLELNVAVNMGEPDNDFFQLRADDLRDETAYRRRIQSAARKLANNVELKVANMAAE
MGSLVITSPDAIGTNTADAWNFVADAEEIMFSRELNRDMGTSYFFNPQDYKKAGYDLTKR
DIFGRIPEEAYRDGTIQRQVAGFDDVLRSPKLPVLTKSTATGITVSGAQSFKPVAWQLDNDG
NKVNVDNRFATVTLSATTGMKRGDKISFAGVKFLGQMAKNVLAQDATFSVVRVVDGTH
VEITPKPVALDDVSLSPEQRAYANVNTSLADAMAVNILNVKDARTNVFWADDAIRIVSQPI
PANHELFAGMKTTSFSIPDVGLNGIFATQGDISTLSGLCRIALWYGVNATRPEAIGVGLPGQ
TA-

Note: The addressable cysteine is labeled in red. The N-terminal extension is underlined. A part of SP (amino acid 239-262) is included in the N-terminal extension (shown in highlight), with GS linkers attached to both sides to increase the overall flexibility of the extension. This design is to mimic the SP$_t$ fusion in the enzymes, which potentially separate the encapsulated enzymes from the capsid shell. The SP$_t$ sequence in CP$_{N-ext}$ does not contain the part of amino acid 263-303, which forms a helix-turn-helix structure, and there are two reasons. 1) This part of SP directs self-assembly of CP.[3] Including this sequence might make CP self-assembly become SP-independent, and prevents SP-directed enzyme encapsulation. 2) The two ends of a helix-turn-helix structure are very close to each other.[4] Thus this structure in the enzyme-SP$_t$ fusion likely does not contribute to the distance between capsid shell and encapsulated enzymes.

CP$_{S39C}$

MALNEGQIVTLAVDEIIETISAITPMAQKAKKYTPPAACMQRSSNTIWMPVEQESPTQEGW
DLTDKATGLLELNVAVNMGEPDNDFFQLRADDLRDETAYRRRIQSAARKLANNVELKVA
NMAAEMGSLVITSPDAIGTNTADAWNFVADAEEIMFSRELNRDMGTSYFFNPQDYKKAG
YDLTKRDIFGRIPEEAYRDGTIQRQVAGFDDVLRSPKLPVLTKSTATGITVSGAQSFKPVAW
QLDNDGNKVNVDNRFATVTLSATTGMKRGDKISFAGVKFLGQMAKNVLAQDATFSVVR
VVDGTHVEITPKPVALDDVSLSPEQRAYANVNTSLADAMAVNILNVKDARTNVFWADDAI
RIVSQPIPANHELFAGMKTTSFSIPDVGLNGIFATQGDISTLSGLCRIALWYGVNATRPEAIG
VGLPGQTA-

Note: The addressable cysteine is labeled in red.

PtDH-SP$_t$

MHHHHHHLPKLVITHRVHEEILQLLAPHCELITNQTDSTLTREEILRRCRDAQAMMAFMP
DRVDADFLQACPELRVIGCALKGFDNFDVDACTARGVWLTFVPDLLTVPTAELAIGLAVG
LGRHLRAADAFVRSGKFRGWQPRFYGTGLDNATVGFLGMGAIGLAMADRLQGWGATL
QYHAAKALDTQTEQRLGLRQVACSELFASSDFILLALPLNADTLHLVNAELLALVRPGAL
LVNPCRGSVVDEAAVLAALERGQLGGYAADVFEMEDWARADRPQQIDPALLAHPNTLFT
PHIGSAVRAVRLEIERCAAQNILQALAGERPINAVNRLPKANPAADTRLSERLTLKPRGKQI
SSAPPADQPITGDVSAANKDAIRKQMDAAASKGDVETYRKLKAKLKGIR-

Note: PtDH sequence is labeled in blue (This PtDH construct was assigned the name 17X-PTDH in

previous publications[5]), while SP_t sequence (amino acid 239-303) is highlighted.

AdhD-SP_t

MGSSHHHHHHSGAKRVNAFNDLKRIGDDKVTAIGMGTWGIGGRETPDYSRDKESIEAIRY
GLELGMNLIDTAEFYGAGHAEEIVGEAIKEFEREDIFIVSKVWPTHFGYEEAKKAARASAK
RLGTYIDLYLLHWPVDDFKKIEETLHALEDLVDEGVIRYIGVSNFNLELLQRSQEVMRKYE
IVANQVKYSVKDRWPETTGLLDYMKREGIALMAYTPLEKGTLARNECLAKIGEKYGKTA
AQVALNYLIWEENVVAIPKASNKEHLKENFGAMGWRLSEEDREMARRCVTRLSERLTLK
PRGKQISSAPHADQPITGDVSAANKDAIRKQMDAAASKGDVETYRKLKAKLKGIR-

Note: AdhD sequence is labeled in green, while SP_t sequence (amino acid 239-303) is highlighted.

ER-SP_t

MSGYHFLKPFTFKHQTITLKNRIVIPPMTTRLSFEDGTVTRDEIRYYQQRAGGVGMFITGT
ANVNALGKGFEGELSVADDRFIPGLSKLAAAMKTGGTKAILQIFSAGRMSNSKILRGEQP
VSASAVAAPRAGYETPRALTSAEIEATIHDFGQAVRRAILAGFDGIELHGANTYLIQQFYSP
NSNRRTDEWGGDRDKRMRFPLAVVHEAEKVIATIADRPFLLGYRISPEELEQPGITLDDTL
ALIDALKQTKIDYLHVSQSDVWRTSLRNPEDTAIMNEQIRDHVAGAFPVIVVGGIKTPADA
EKAAESFDLVAIGHEMIREPHWVQKVLDHDEKAIRYQIAPADLEELGIAPTFLDFIESISGG
AKGVPLTTAQSVTSSNVTQDAGSLVPRGSARSNAVAEQGRKTQEFTQQSAQYVEAARKH
YDAAEKLNIPDYQEKEDAFMQLVPPAVGADIMRLFPEKSAALMYHLGANPEKARQLLAM
DGQSALIELTRLSERLTLKPRGKQISSAPPADQPITGDVSAANKDAIRKQMDAAASKGDVE
TYRKLKAKLKGIRLVGHHHHHH-

Note: ER sequence is labeled in turquois, while SP_t sequence (amino acid 141-303) is highlighted.

References

(1) Gao, X.; Ren, J.; Wu, Q.; Zhu, D. Biochemical characterization and substrate profiling of a new NADH-dependent enoate reductase from Lactobacillus casei. *Enzyme Microb Technol* **2012**, *51* (1), 26-34. DOI: 10.1016/j.enzmictec.2012.03.009.

(2) Selivanovitch, E.; LaFrance, B.; Douglas, T. Molecular exclusion limits for diffusion across a porous capsid. *Nat Commun* **2021**, *12* (1), 2903. DOI: 10.1038/s41467-021-23200-1.

(3) Weigele, P. R.; Sampson, L.; Winn-Stapley, D.; Casjens, S. R. Molecular genetics of bacteriophage P22 scaffolding protein's functional domains. *J Mol Biol* **2005**, *348* (4), 831-844. DOI: 10.1016/j.jmb.2005.03.004.

(4) (a) Chen, D. H.; Baker, M. L.; Hryc, C. F.; DiMaio, F.; Jakana, J.; Wu, W.; Dougherty, M.; Haase-Pettingell, C.; Schmid, M. F.; Jiang, W.; et al. Structural basis for scaffolding-mediated assembly and maturation of a dsDNA virus. *Proc Natl Acad Sci U S A* **2011**, *108* (4), 1355-1360. DOI: 10.1073/pnas.1015739108. (b) Sun, Y.; Parker, M. H.; Weigele, P.; Casjens, S.; Prevelige, P. E.; Krishna, N. R. Structure of the coat protein-binding domain of the scaffolding protein from a double-stranded DNA virus. *J Mol Biol* **2000**, *297* (5), 1195-1202. DOI: 10.1006/jmbi.2000.3620.

(5) Hung, J. E.; Fogle, E. J.; Christman, H. D.; Johannes, T. W.; Zhao, H.; Metcalf, W. W.; van der Donk, W. A. Investigation of the role of Arg301 identified in the X-ray structure of phosphite dehydrogenase. *Biochemistry* **2012**, *51* (21), 4254-4262. DOI: 10.1021/bi201691w.
